# Effects of transcranial magnetic stimulation on spatial memory: a systematic review across animal and human studies

**DOI:** 10.3389/fnbeh.2026.1901070

**Published:** 2026-08-03

**Authors:** Julia C. Lobo, Alba Gutiérrez-Menéndez, Marta Mendez, Candela Zorzo

**Affiliations:** 1 Department of Psychology, Faculty of Psychology, University of Oviedo, Oviedo, Spain; 2 Instituto de Neurociencias del Principado de Asturias, Oviedo, Spain; 3 Instituto de Investigacion Sanitaria del Principado de Asturias, Oviedo, Spain; 4 Universidad Internacional de Valencia, Valencia, Spain; 5 Universidad Internacional de la Rioja, Logroño, Spain

**Keywords:** brain stimulation, memory, neuromodulation, spatial learning, transcranial magnetic stimulation, hippocampus, synaptic plasticity

## Abstract

**Introduction:**

Spatial memory relies on distributed hippocampal–cortical networks that are highly sensitive to modulation of excitability, connectivity, and synaptic plasticity. Transcranial magnetic stimulation (TMS) has emerged as a promising tool to experimentally probe and therapeutically modulate these networks, although its effects on spatial memory remain heterogeneous.

**Methods:**

We conducted a PRISMA-guided systematic review synthesizing evidence from rodent and human studies examining the effects of TMS on spatial memory. A total of 35 studies (23 animal, 12 human) identified through searches of Scopus, Web of Science, and PubMed were included, encompassing a broad range of stimulation protocols, behavioral paradigms, and neurophysiological outcomes.

**Results:**

In animal models, TMS consistently improved spatial learning and memory under pathological conditions, including neurodegenerative, vascular, stress-related, and injury models. These effects were associated with convergent mechanisms, including restoration of hippocampal long- term potentiation, modulation of neurotrophic signaling, reduced apoptosis and neuroinflammation, enhanced synaptic plasticity, and recovery of hippocampal network function. In contrast, findings in healthy animals were mixed and strongly dependent on stimulation parameters. Human findings were similarly variable. Improvements were observed in some clinical populations, whereas results in healthy individuals were inconsistent and task dependent. Neurophysiological evidence indicated modulation of oscillatory activity and hippocampal–cortical connectivity, although these changes did not always translate into measurable behavioral effects.

**Discussion:**

Overall, TMS effects on spatial memory appear to be strongly state-dependent, reflecting interactions between stimulation parameters and underlying circuit integrity. Further research should prioritize multimodal approaches integrating behavioral and neurophysiological measures, particularly in clinical populations, to improve mechanistic understanding and translational applicability.

**Systematic review registration:**

https://osf.io/, identifier 10.17605/OSF.IO/32VWN.

## 1 Introduction

Spatial memory is a core cognitive function that enables humans and other animals to encode, maintain, and retrieve information about spatial relationships and locations (Tommasi and Laeng, 2012), thereby supporting adaptive behavior in dynamic and changing environments (Epstein et al., 2017). The neurobiological bases of spatial memory have been extensively characterized and are supported by an extended hippocampal-diencephalic-cortical network, encompassing the hippocampal formation, parahippocampal and retrosplenial cortices, thalamic nuclei (particularly the anterior thalamus), posterior parietal regions, and prefrontal areas (Todd and Bucci, 2015; Eichenbaum, 2017; Ranganath, 2019; Ekstrom and Hill, 2023). In parallel, synaptic mechanisms, including long-term potentiation (LTP), early consolidation processes, and activity-dependent plasticity, contribute to the stabilization and long-term maintenance of spatial memories (Kandel et al., 2014; Dudai et al., 2015; Asok et al., 2019; Goto, 2022).

Given the distributed architecture of spatial memory and the mechanisms required for its long-term stabilization, it is particularly vulnerable to alterations in neuronal excitability, inter-regional connectivity, and synaptic plasticity (Broadbent et al., 2006; Cipolotti et al., 2008; Vann et al., 2009; Wartman et al., 2014; Kim et al., 2018; Shen et al., 2025). Disruptions across hippocampal, thalamic, retrosplenial, or prefrontal nodes, whether due to aging, neurodegenerative disease, brain injury, or psychiatric and psychological conditions, can compromise the encoding and retrieval of spatial information and lead to pronounced deficits in spatial cognition (Spieker et al., 2012; Gallagher et al., 2015; An et al., 2016; Yu et al., 2018; Merhav et al., 2019; Kim et al., 2020; Wang et al., 2020; Silva and Martínez, 2022; García-Navarra et al., 2025). Thus, understanding how these networks can be modulated is a key question in cognitive neuroscience and clinical research. Spatial memory was selected as the focus of this review not because it is considered more relevant than other cognitive domains, but because its hippocampal- cortical substrate is particularly vulnerable to early pathological change, as the hippocampus is among the first structures affected in both neurodegenerative (Braak and Braak, 1991; Rao et al., 2022) and vascular/ischemic conditions (Schmidt-Kastner, 2015).

In this context, non-invasive brain stimulation techniques have emerged as promising approaches to modulate the distributed cortical-subcortical networks that support spatial memory (England et al., 2015; Shang et al., 2016; Zorzo et al., 2021), particularly when these circuits are disrupted by neurological or psychological conditions (de Sousa et al., 2020), offering a more broadly applicable alternative to invasive approaches such as deep brain stimulation, which require surgical electrode implantation and are typically reserved for severe, treatment-refractory conditions. Among them, transcranial magnetic stimulation (TMS) enables controlled modulation of cortical excitability and network dynamics (Klomjai et al., 2015; Jannati et al., 2023), offering a unique opportunity to influence hippocampal-dependent spatial memory functions. By delivering brief magnetic pulses that induce electric fields in the cortex, TMS can alter ongoing neural activity and propagate changes across interconnected brain networks (Rossini et al., 2015; Hallett et al., 2017; Sydnor et al., 2022). Notably, depending on the stimulation parameters, it can transiently enhance or suppress cortical activity and induce short- or long-lasting plasticity effects (Klomjai et al., 2015; Chen et al., 2023), with recent evidence further indicating that inhibitory and excitatory rTMS protocols produce distinct, layer-specific microstructural changes within stimulated cortical regions (Zhang et al., 2023).

Altogether, these network-level effects position TMS as a promising neuromodulatory tool to counteract dysfunction within cortical-hippocampal circuits and potentially enhance spatial memory performance in clinical and pathological conditions. This translational potential is increasingly supported by emerging evidence in psychiatric populations, where neuroimaging biomarkers are being used to guide individualized TMS targeting and improve treatment response, such as functional-connectivity-guided target selection in depression (Gao et al., 2025) and neuroimaging-informed treatment planning in schizophrenia (Zhang W. et al., 2025).

Over the past decades, TMS has been employed in diverse animal models and human populations to investigate its effects on spatial cognition. In rodent studies, stimulation is typically delivered over scalp locations that indirectly engage hippocampal and related circuits, both in healthy animals (Ma et al., 2014; Shang et al., 2016) and in neuropathological models, including Alzheimer’s disease–related models (Tan et al., 2013; Bao et al., 2021), vascular and ischemic injury models (Yang et al., 2015) and stress- or sleep deprivation–related paradigms (Shang et al., 2019; Zheng et al., 2024).

**FIGURE 1:**
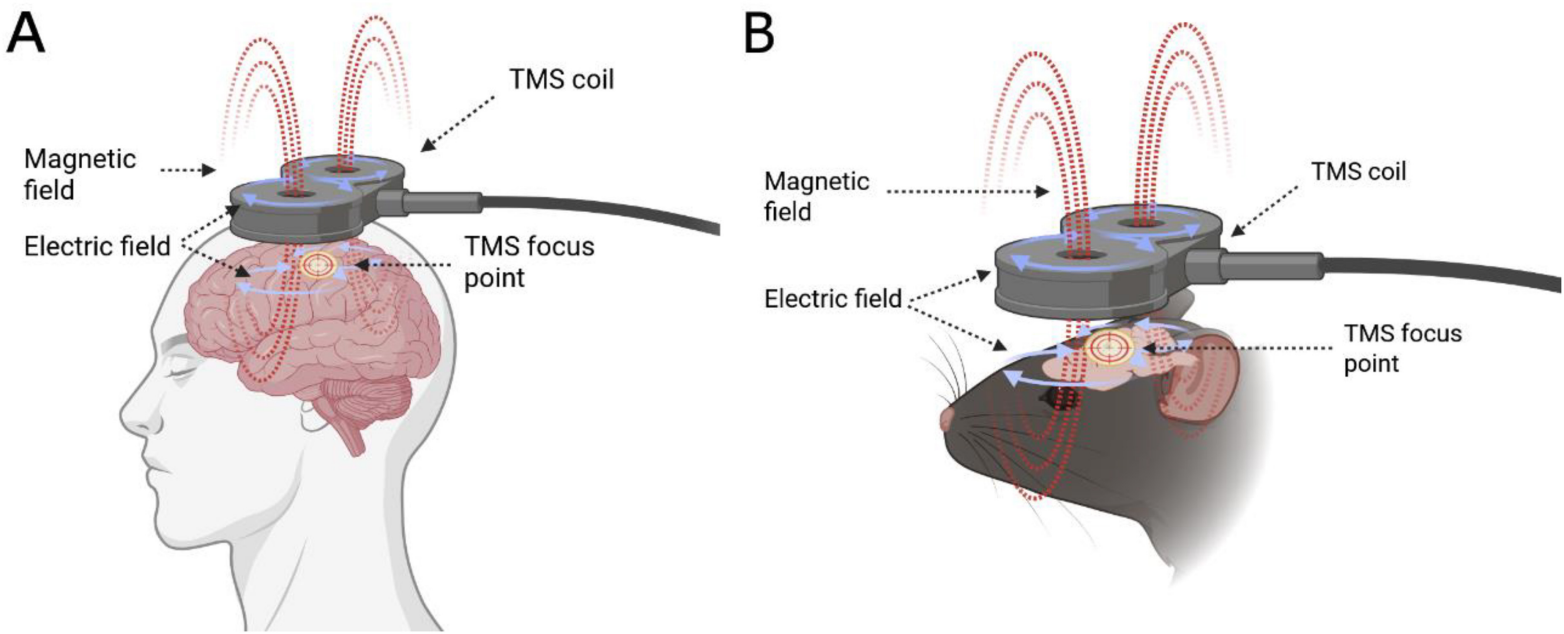
Two labeled illustrations compare transcranial magnetic stimulation (TMS) on a human **(A)** and a rodent **(B)**, displaying TMS coils, magnetic fields, electric fields, and stimulation focus points within detailed brain cross-sections. Created with BioRender.com.

In humans, TMS has similarly been applied in healthy individuals (Guidali et al., 2019; Deng et al., 2024), as well as in clinical populations, including psychiatric disorders (Demirtas-Tatlidede et al., 2010; Asl et al., 2024) and Alzheimer’s disease (Traikapi et al., 2023), to examine how modulation of large-scale networks influences spatial cognitive processes. Despite this growing interest, the literature shows substantial variability in stimulation parameters (e.g., coil type, frequency, intensity, number of pulses, stimulation pattern, and target brain areas) as well as in spatial memory outcome measures. As a result, direct comparisons across studies are difficult, limiting firm conclusions about how specific TMS protocols modulate spatial memory–related circuits and behavior across species. A comparative illustration of representative TMS configurations in human and rodent participants is provided in Figure 1.

Previous reviews have mostly addressed broader cognitive domains (Begemann et al., 2020), clinical applications of TMS (Burke et al., 2013; Xiao et al., 2024), or its neurophysiological mechanisms (Klomjai et al., 2015; Jannati et al., 2023), but none has systematically focused on spatial memory, nor integrated evidence from both human and rodent work. The objective of this systematic review is therefore to identify, evaluate and synthesize all original experimental studies investigating the effects of TMS on spatial memory in humans and rodent models. By systematically examining stimulation parameters, task paradigms, behavioral or cognitive outcomes, and neurophysiological effects, this review aims to provide an integrated framework for interpreting current findings and to outline evidence-based recommendations for future research on TMS modulation of spatial memory.

## 2 Methods

### 2.1 Search strategy

The search and study selection adhered to the Preferred Reporting Items for Systematic Review and Meta-Analysis guidelines (PRISMA) 2020 statement (Page et al., 2021). The review was registered in the Open Science Framework (registration ID: 10.17605/OSF.IO/32VWN).

A comprehensive search was conducted in Scopus, Web of Science (WoS), and PubMed databases on September 29th, 2025, with no date restriction. The search was limited to original research articles and applied a combination of terms (MeSH terms). The following Boolean combination was used: (“transcranial magnetic stimulation” OR “repetitive transcranial magnetic stimulation” OR “TMS” OR “theta burst stimulation”) AND (“spatial memory” OR “spatial working memory” OR “spatial short-term memory” OR “spatial long-term memory” OR “spatial recovery” OR “spatial recall”) NOT (“review”).

### 2.2 Selection criteria

The articles were selected according to the following inclusion criteria: (a) original, peer- reviewed empirical studies reporting experimental data; (b) studies examining the effects of TMS, including any stimulation protocol (single-pulse, paired-pulse, repetitive TMS (rTMS), or patterned protocols such as theta burst stimulation (TBS), including intermittent TBS (iTBS) or continuous TBS (cTBS)); (c) human studies involving healthy or clinical populations; (d) rodent studies using healthy or pathological models in rats or mice; and (e) assessment of spatial memory. Only studies reporting quantitative spatial memory outcomes after TMS were included.

Articles that did not meet these criteria and/or met the following exclusion criteria were not included: (a) non-empirical or non-original publications (reviews, case reports, conference papers, editorials, letters, dissertations, and commentaries); (b) studies involving in vitro, computational, or technical simulations without in vivo cognitive assessment; (c) studies assessing only general memory, motor outcomes, executive functions, or other cognitive domains without a spatial component; (d) qualitative or descriptive studies; (e) articles published in languages other than English.

### 2.3 Screening for inclusion and study selection

The initial search was conducted across Scopus, WoS, and PubMed databases, producing 356, 227, and 301 records, respectively, which amounted to a combined total of 884 references. Duplicate entries were first identified through Mendeley and subsequently verified manually. After removing the duplicates, the dataset was reduced to 583 unique articles. These records were screened independently by two reviewers, with a third reviewer checking for consistency and resolving discrepancies.

**FIGURE 2:**
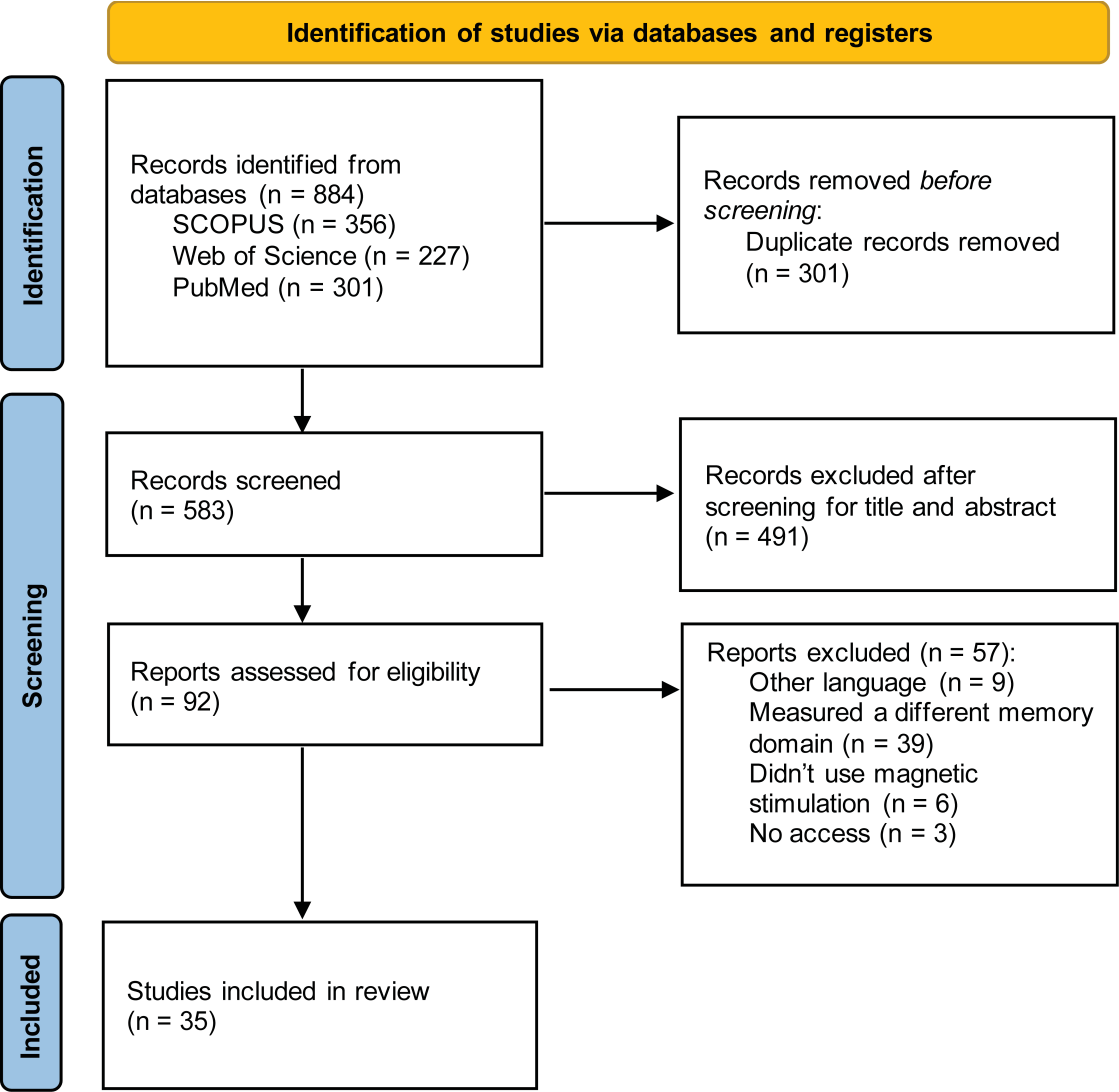
Flowchart illustrating the PRISMA method for study selection, showing an initial 884 records identified, duplicates removed, 583 records screened, 92 reports assessed, and 35 studies included in the review. Key exclusion reasons are provided.

Titles and abstracts were examined to exclude studies that did not satisfy the aforementioned inclusion criteria, resulting in 92 eligible articles for full-text review. Full texts were then assessed, and studies that failed to meet the criteria or were inaccessible were excluded, leaving a final sample of 35 studies. The complete selection process workflow is depicted in Figure 2.

### 2.4 Quality assessment and risk of bias

The methodological quality and risk of bias of both animal and human studies were assessed using the SYRCLE Risk of Bias tool and the Joanna Briggs Institute (JBI) Critical Appraisal Checklists, respectively (Hooijmans et al., 2014; Barker et al., 2023; Aromataris et al., 2024), selecting the appropriate tool according to study design. Two independent reviewers conducted the assessment, and discrepancies were resolved through discussion. For the human studies, the appropriate JBI checklist was selected according to the study design: randomized controlled trials (RCTs, 13 items) and quasi- experimental studies (nine items). Items were rated as “Yes,” “No,” “Unclear,” or “Not applicable,” with higher scores indicating greater methodological quality. Animal studies were assessed using the SYRCLE risk-of-bias tool, covering selection, performance, detection, attrition, reporting, and other bias domains. Each domain was rated as “low risk” (L), “high risk” (H), or “unclear” (U).

Overall, the quality assessment indicated that the human studies were of adequate methodological quality, showing low-to-moderate risk of bias, while the animal studies suggest an acceptable methodological quality, albeit with an unclear risk of bias. A detailed summary of the individual scores and criteria is provided in Table 1.

**TABLE 1:** Risk of bias using the JBI critical appraisal checklist for human studies, and SYRCLE risk of bias tool for animal studies.

References	Q1	Q2	Q3	Q4	Q5	Q6	Q7	Q8	Q9	Q10	Q11	Q12	Q13	Overall score
Human RCTs														
Asl et al., 2024	Y	U	Y	U	U	Y	Y	Y	U	U	N	Y	Y	7
Deng et al., 2024	Y	U	Y	Y	U	Y	U	Y	U	Y	N	Y	Y	8
Dordevic et al., 2022	Y	U	N/A	U	U	Y	U	Y	U	U	N/A	Y	Y	5
Hermiller et al., 2022	Y	U	N/A	U	U	Y	U	Y	U	U	U	Y	Y	5
Huang et al., 2024	U	U	N/A	Y	U	Y	U	Y	Y	Y	N	Y	Y	6
Marin et al., 2018	Y	U	Y	U	U	Y	U	Y	U	Y	N	Y	Y	7
Tang et al., 2023	Y	Y	N	Y	U	Y	Y	Y	U	Y	N	Y	Y	9
Human quasi-experimental Studies														
Demirtas-Tatlidede et al., 2010	Y	N	N/A	N/A	Y	Y	U	Y	Y	–	–	–	–	–
Goldsworthy et al., 2020	Y	Y	N	Y	Y	Y	U	U	Y	–	–	–	–	–
Guidali et al., 2019	Y	Y	Y	Y	Y	Y	Y	Y	Y	–	–	–	–	–
Nilakantan et al., 2017	Y	Y	Y	Y	Y	Y	U	Y	Y	–	–	–	–	–
Traikapi et al., 2023	Y	Y	Y	Y	Y	Y	Y	Y	Y	–	–	–	–	–
Animal experimental studies														
Bao et al., 2021	L	L	U	L	L	U	L	L	L	L	–	–	–	–
Hong et al., 2024	L	L	U	L	U	U	U	L	L	L	–	–	–	–
Huang et al., 2017	L	L	U	L	L	U	L	L	L	L	–	–	–	–
Jiang et al., 2023	L	L	U	L	L	U	U	L	L	L	–	–	–	–
Jiang et al., 2025	L	L	U	L	U	U	U	L	L	L	–	–	–	–
Kim et al., 2024	L	L	U	L	U	U	U	L	L	L	–	–	–	–
Li et al., 2007	U	L	U	L	U	U	U	L	L	L	–	–	–	–
Lin et al., 2024	L	L	U	L	U	U	L	L	L	L	–	–	–	–
Ma et al., 2014	L	L	U	L	U	L	U	L	L	L	–	–	–	–
Ma et al., 2017	L	L	U	L	U	L	U	L	L	L	–	–	–	–
Ma et al., 2019	L	L	U	L	U	U	U	L	L	L	–	–	–	–
Qin et al., 2024	L	L	U	L	U	L	U	L	L	L	–	–	–	–
Shang et al., 2016	U	L	U	L	U	U	U	L	L	L	–	–	–	–
Shang et al., 2019	L	L	U	L	U	U	U	L	L	L	–	–	–	–
Tan et al., 2013	L	L	U	L	L	L	U	L	L	L	–	–	–	–
Wang et al., 2015	U	L	U	L	L	U	L	L	L	L	–	–	–	–
Wu et al., 2022	L	L	U	U	U	U	U	L	L	L	–	–	–	–
Yang et al., 2014	L	L	U	U	U	L	L	L	L	U	–	–	–	–
Yang et al., 2015	L	L	U	L	U	U	U	L	L	U	–	–	–	–
Zhang et al., 2015	L	L	U	L	U	U	U	L	L	U	–	–	–	–
Zhang et al., 2024	L	L	U	L	U	U	U	L	L	L	–	–	–	–
Zheng et al., 2024	L	L	U	L	U	U	U	L	L	L	–	–	–	–
Zorzo et al., 2021	L	L	U	L	U	U	L	L	L	L	–	–	–	–

Y, yes, U, unclear, N, no; N/A, not applicable; L, low risk; H, high risk. JBI questions for human RCT studies: Q1, Was true randomization used for assignment of participants to treatment groups?; Q2, Was allocation to treatment groups concealed?; Q3, Were treatment groups similar at the baseline?; Q4, Were participants blind to treatment assignment?; Q5, Were those delivering the treatment blind to treatment assignment?; Q6, Were treatment groups treated identically other than the intervention of interest?; Q7, Were outcome assessors blind to treatment assignment?; Q8, Were outcomes measured in the same way for treatment groups?; Q9, Were outcomes measured in a reliable way?; Q10, Was follow up complete and if not, were differences between groups in terms of their follow up adequately described and analysed?; Q11, Were participants analysed in the groups to which they were randomized?; Q12, Was appropriate statistical analysis used?; Q13, Was the trial design appropriate and any deviations from the standard RCT design (individual randomization, parallel groups) accounted for in the conduct and analysis of the trial?. JBI questions for quasi-experimental human studies: Q1, Is it clear in the study what is the “cause” and what is the “effect” (i.e. there is no confusion about which variable comes first)?; Q2, Was there a control group?; Q3, Were participants included in any comparisons similar?; Q4, Were the participants included in any comparisons receiving similar treatment/care, other than the exposure or intervention of interest?; Q5, Were there multiple measurements of the outcome, both pre and post the intervention/exposure?; Q6, Were the outcomes of participants included in any comparisons measured in the same way?; Q7, Were outcomes measured in a reliable way?; Q8, Was follow-up complete and if not, were differences between groups in terms of their follow-up adequately described and analysed?; Q9, Was appropriate statistical analysis used?. SYRCLE questions for experimental animal studies: Q1, Was the allocation sequence adequately generated and applied?; Q2, Were the groups similar at baseline or were they adjusted for confounders in the analysis?; Q3, Was the allocation adequately concealed?; Q4, Were the animals randomly housed during the experiment?; Q5, Were the caregivers and/or investigators blinded from knowledge of which intervention each animal received during the experiment?; Q6, Were animals selected at random for outcome assessment?; Q7, Was the outcome assessor blinded?; Q8, Were incomplete outcome data adequately addressed?; Q9, Are reports of the study free of selective outcome reporting?; Q10, Was the study apparently free of other problems that could result in high risk of bias?

## 3 Results

This review comprises a total of 35 studies displaying considerable methodological heterogeneity in terms of sample characteristics, pathological or experimental models, stimulation protocols, behavioral tasks and neurobiological outcome measures. Due to the significant differences between animal and human research, the results are presented separately. The impact of TMS on spatial memory and brain outcomes is presented in Table 2 (rodent) and Table 3 (human), including sample characteristics. Stimulation parameters are presented in Table 4 (rodent) and Table 5 (human). Overall, 23 animal studies (65.7%) and 12 human studies (34.3%) were included.

**TABLE 2:** Characteristics and main outcomes of rodent studies examining the effects of TMS on spatial memory.

References	Sample characteristics: animal/sex/age/ model	Total sample size/groups (*n*)	Anesthetized/immobilization	Memory domain/ apparatus	Brain measures	Memory outcomes	Brain outcomes
Bao et al., 2021	Mice/male/6 months/ /Alzheimer’s disease (SAMP8)	*N* = 60/control (*n* = 20), SAMP8 (*n* = 20), rTMS + SAMP8 (*n* = 20)	NR/NR	Spatial memory/ MWM	HE; TUNEL assay; IHC (Caspase-3, Bax and Bcl-2); qRT-PCR (Caspase-3, Bax and Bcl-2); WB (cAMP, PKAc and p-CREB)	rTMS improved spatial learning and memory deficits (reduced escape latency, increased target quadrant crossings)	rTMS reduced neuronal degeneration, necrosis, the number of apoptotic neurons and cell loss in cortex and hippocampus; decreased Bax and increased Bcl-2 in both regions and reduced Caspase-3 in cortex; decreased Bax and Caspase- 3 mRNA and increased Bcl- 2 mRNA in cortex and hippocampus; and increased cAMP, PKAc and p-CREB protein levels in both regions.
Hong et al., 2024	Rat/male/7 weeks/Stroke (tMCAO)	*N* = 30/Sham (*n* = 10), tMCAO (*n* = 10), tMCAO + rTMS (*n* = 10)	None/restrained in bags	Spatial memory/ MWM	TEM (synaptic ultrastructure); WB (PSD95, SYN and BDNF)	rTMS improved spatial learning and memory (reduced escape latency and increased platform crossings)	rTMS restored hippocampal synaptic ultrastructure (length of active zone, width of synaptic cleft and PSD thickness) and increased PSD95, SYN and BDNF expression in the hippocampus.
Huang et al., 2017	Mice/male/1.5 months/ Alzheimer’s disease (APP23/PS45)	*N* = 57 (MWM, IHC and WB)/ WT Sham (*n* = 16), WT + rTMS (*n* = 13), AD Sham (*n* = 15), AD + rTMS (*n* = 13); *N* = 21 (electrophysiology)/ WT Sham (*n* = 6), WT + rTMS (*n* = 3), AD Sham (*n* = 7), AD + rTMS (*n* = 5)	Anesthetized in preliminary MT determination/ Restrained in cloth sleeve	Spatial memory/ MWM	Electrophysiolo gy (hippocampal LTP); IHC (Aβ plaques); WB (APP, C99, C89 and BACE1)	rTMS improved spatial learning and memory (reduced escape latency, increased target quadrant time and platform crossings)	rTMS restored hippocampal LTP and reduced amyloidogenic processing of APP (decreased BACE1, C99 and C89) and Aβ plaque load.
Jiang et al., 2023	Rat/male/adults/ depression (CUMS)	*N* = 32/control (*n* = 8), CUMS + Sham (*n* = 8), CUMS (*n* = 8), CUMS + rTMS (*n* = 8)	None/head fixed	Shortterm spatial memory/ Y-maze	IF (nestin and GFAP); WB (nestin and GFAP)	rTMS reversed the CUMS-induced impairment in spatial memory (novel arm preference)	rTMS restored CUMS- induced alterations in hippocampal CA3 GFzAP and nestin expression
Jiang et al., 2025	Rat/Male/8 weeks/ stroke (MCAO/R)	*N* = 100 (exp. 3)/ Sham + LV-control (*n* = 25), MCAO + LV-control (*n* = 25), MCAO + TMS + LV-control (*n* = 25), MCAO + TMS +LV-SARM1 (*n* = 25)	Anesthetized/none	Spatial memory/ MWM	TCC; WB (SARM1, HSP70, NF-κB, Bax, Bcl-2, GAP-43, NF- 200, BDNF and GAPDH), IF (SARM1, NeuN and MBP); FJB; IHC (GAP-43 and NF-200); HE; TEM (myelination)	TMS hastened spatial learning and increased target quadrant time and distance of MCAO/R + LV- control rat	TMS mitigated hippocampal cell apoptosis (fostered hippocampal axons and myelin sheaths regeneration) via SARM1/ HSP70/ NF-κB signaling pathway
Kim et al., 2024	Rat/male/10 weeks/ traumatic brain injury (DAI)	*N* = 18/ DAI (*n* = 9), DAI + rTMS (*n* = 9)	Anesthetized/none	Spatial memory/ MWM	WB (BDNF, VEGF, and MAP2)	rTMS reduced escape latencies	rTMS elevated expression levels of BDNF, VEGF, and MAP2 in the hippocampus
Li et al., 2007	Rat/male/10–12 weeks/healthy	*N* = 28/Sham (*n* = 7), Chronic rTMS (*n* = 7), Sham (*n* = 7), acute rTMS (*n* = 7)	NR/NR	Spatial memory/ MWM	None	Chronic rTMS impaired short- and long-term spatial reference memory; acute rTMS impaired only longterm spatial reference memory; acquisition was unaffected.	None
Lin et al., 2024	Mice/male/8–10 weeks/cerebral microinfarct	*N* = 32/ Control (*n* = 8), Control + cTBS (*n* = 8), microinfarct (*n* = 8), microinfarct + cTBS (*n* = 8)	NR/NR	Spatial memory/ MWM	Two-photon microscopy (synaptic ultrastructure); IF (AQP4, NeuN, GFAP and Ibal); CSF-ISF tracer imaging (FITC- dextran and rhodamine B)	cTBS attenuated spatial memory impairment (reduced escape latency and increased target quadrant time)	cTBS enhanced paravascular CSF-ISF exchange and CSF infiltration into brain parenchyma; restored AQP4 expression and perivascular polarization, attenuated neuronal loss, and reduced astrocytic and microglial activation in lesion areas
Ma et al., 2014	Mice/Male/15 months (aged) and 6 months (adult)/ normal aging	*N* = 96/ aging-control (*n* = 24), aging-sham (*n* = 24), aging-LIMS (110% RMT, *n* = 24), aging-HIMS (150% RMT, *n* = 24)	Anesthetized in MT determination/ restrained in cloth sleeve	Spatial memory/ MWM	TEM (neuronal and synaptic ultrastructure); IHC (SYN, GAP-43, and PSD95); WB and Semi RT- PCR/ qRT-PCR (SYN, GAP-43, PSD95, BDNF and TrkB)	rTMS (110% RMT) improved spatial learning and memory (reduced escape latency and increased target quadrant time and platform crossings), whereas rTMS (150% RMT) worsened spatial memory performance	rTMS (110% RMT) enhanced hippocampal synaptic ultrastructure (increased synapse density and PSD thickness); up- regulated SYN, GAP-43 and PSD95 at protein and mRNA levels; and activated BDNF-TrkB signaling. rTMS (150% RMT) induced synaptic ultrastructural degeneration (decreased synapse density, PSD thinning, lipofuscin accumulation); down- regulated SYN, GAP-43, PSD95 and BDNF-TrkB expression; and showed ultrastructural features of neuronal damage/apoptosis
Ma et al., 2017	Mice/male/7 months/ senescence-aged (SAMP8)	*N* = 60/ SAMR1 control (*n* = 20), SAMP8-sham (*n* = 20), SAMP8-rIMS (*n* = 20)	None/restrained in flexible plastic cylindrical mold	Spatial memory/MWM	WB (SYN and PSD95); RT- PCR (SYN and PSD95)	rTMS attenuated spatial memory impairment in SAMP8 mice (reduced escape latency and increased platform crossings), although performance did not fully reach SAMR1 control levels	rTMS up-regulated SYN and PSD95 at protein and mRNA levels in the hippocampus
Ma et al., 2019	Mice/male/15 months (aged) and 3 months (adult)/normal aging	*N* = 60/ Aged-sham (*n* = 15), Adult-sham (*n* = 15), 5 Hz rTMS (*n* = 15), 25 Hz rTMS (*n* = 15)	None/restrained in flexible plastic cylindrical mold	Spatial memory/MWM	TEM (synaptic ultrastructure); WB (SYN, PSD95, BDNP and pCREB); gene expression profiling (synaptic plasticity- related genes)	rTMS attenuated spatial memory impairment (reduced escape latency and increased platform crossings), with greater improvement at 5 Hz than 25 Hz	5 Hz rTMS increased PSD thickness and reduced synaptic cleft width; up- regulated SYN and PSD95; activated BDNF-pCREB signaling; gene expression profiling showed upregulation of neuroplasticity-related genes (BDNF, AC1, PSD95, CaMKII): and downregulation of PPI.
Qin et al., 2024	Mice/male/6–8 weeks/brain injury (RIBI)	*N* = 36/ Sham (*n* = 12), cranial irradiation (*n* = 12), rTMS (*n* = 12)	None/immobilized in fixator	Spatial memory/MWM	IF (DCX, Ki67, BrdU, Iba-1 and GSDMD); IHC (Iba-1); WB (BDNF, p- TrkB/TrkB, p- CREB/CREB, NLRP3, ASC, Caspase-1, GSDMD-N, IL-1β, PSD95 and SYN); Golgi staining (dendritic spine density)	rTMS improved spatial learning and memory performance compared with irradiated mice. Blockade of BDNF signaling with K252a reversed the rTMS-induced cognitive improvement (increased escape latency and reduced target quadrant time)	rTMS promoted hippocampal neurogenesis (increased DCX, Ki67, BrdU+/DCX+ and BrdU+/NeuN+ cells); attenuated microglial activation and pyroptosis (decreased Iba-1+ and GSDMD+/Iba-1+ cells); downregulated NLRP3 inflammasome components (reduced NLRP3, ASC, Caspase-1, GSDMD-N, IL-1β). rTMS activated BDNF signaling (elevated BDNF, p-TrkB/TrkB and p- CREB/CREB) and enhanced synaptic plasticity (increased PSD95, SYN, dendritic spine density in DG). All these effects were reversed by BDNF pathway inhibition with K252a
Shang et al., 2016	Rat/male/ adult/healthy	*N* = 18/ Sham (*n* = 6), rTMS (*n* = 6), rTMS without behavioral tasks (*n* = 6)	None/restrained manually during stimulation	Spatial memory/MWM	*In vivo* electrophysiolo gy (LTP, STP, DEP and PPF); WB (BDNF, SYN and NR2B)	rTMS improved spatial learning and memory (reduced escape latency, increased target quadrant time and platform crossings)	rTMS enhanced hippocampal synaptic plasticity (increased STP, LTP, DEP, PPF in CA1) and upregulated hippocampal BDNF, presynaptic SYN and postsynaptic NR2B protein levels. No differences were observed between the rTMS and rTMS without behavior
Shang et al., 2019	Rat/male/NR/stress (PNS)	*N* = 36/ Control (*n* = 8), PNS (*n* = 8), PNS + rTMS (*n* = 8), PNS + rTMS + DMSO (*n* = 6), PNS + rTMS + K252a (*n* = 6)	None/restrained manually during stimulation	Spatial memory/ MWM	*In vivo* electrophysiolo gy (LTP from Schaffer collaterals to CA1); WB (BDNF, TrkB, p-CREB/CREB, NR2A, NR2B, SYN, Bcl-xL, Bax and Caspase-3); IF (NR2A and NR2B); HE (cell number in CA1)	rTMS ameliorated PNS-induced spatial learning and reference memory deficits (reduced escape latency and path length, increased cognitive strategy scores, target quadrant dwell time and platform crossings). These cognitive improvements were abolished by BDNF pathway inhibition with K252a	rTMS enhanced hippocampal synaptic plasticity (increased LTP); up-regulated BDNF/TrkB and p-CREB/CREB signaling; increased NR2A and NR2B expression; preserved neuronal cell number in CA1; and prevented apoptosis (elevated Bcl-xL/ Bax ratio and decreased Caspase-3). All these effects were reversed by K252a
Tan et al., 2013	Rat/male/2.5 months/ Alzheimer’s disease (Aβ_1–42_-induced)	*N* = 84/ Control (*n* = 21), Control + rTMS (*n* = 21), Aβ (*n* = 21), Aβ + rTMS (*n* = 21)	None/restrained in cloth sleeve	Spatial memory/ MWM	IHC (Aβ_1–42_); *in vivo* electrophysiology (LTP); WB (NR1, NR2A and NR2B); ELISA (BDNF and NGF)	rTMS rescued Aβ_1–42_-induced spatial memory deficits (reduced escape latency and swimming distance and increased target quadrant time). rTMS also enhanced memory retrieval in control rats	rTMS partially restored Aβ_1–42_-suppressed hippocampal LTP in the perforant path-DG pathway; up-regulated hippocampal NMD A receptor subunits (increased NR1, NR2A, NR2B), and enhanced hippocampal neurotrophins levels (BDNF, NGF)
Wang et al., 2015	Mice/male/4–5 months/Alzheimer’s disease (3xTg model)	N, not applicable/3xTg sham (*n* = 8), 3xTg 1 Hz (*n* = 7), 3xTg 10 Hz (*n* = 10), 3xTg 15 Hz (*n* = 13); N, not applicable/WT sham (*n* = 9), WT 1 Hz (NR), WT 10 Hz (*n* = 6), WT15 Hz (*n* = 10)	None/restrained manually during stimulation with a plastic cylinder	Spatial memory/ MWM	*Ex vivo* electrophysiolo gy (hippocampal CA1 LTP); whole-cell patch-clamp recordings in cingulate cortex pyramidal neurons (neuronal excitability and BK channel activity); qRT- PCR (BK channel, Homer1a and Homer1c mRNA); ELISA (Aβ_1–42_)	IMS ameliorated spatial learning deficits in a frequencydependent manner (reduced escape latency and increased target quadrant time in the 15 Hz stimulation group, but not at other frequencies)	15 Hz IMS restored the activity of BK channels; enhanced hippocampal longterm potentiation; and modulated cortical excitability. These effects were associated with increased expression of the activity-dependent scaffold protein Homer1a and reduced Aβ levels
Wu et al., 2022	Rat/male/adult/healthy	*N* = 30/sham rTMS, (*n* = 15), rTMS (*n* = 15)	None/restrained in cloth sleeve	Spatial memory/ MWM	rs-fMRI (ReHo, DMN and corti co-striatal- thalamic network activity); WB (PFC, hippocampus andMl:NRl, NR2A, NR2B, BDNF, TrkB, Akt and p-Akt)	rTMS over PFC enhanced spatial episodic learning and memory (reduced escape latency, increased platform crossings and increased target quadrant time)	rTMS increased ReHo in PFC, hippocampus, M1/M2 and other DMN and corti co- striatal-thalamic regions; up-regulated NMD A receptor subunits (NR1, NR2A, NR2B) and BDNF/TrkB/p-Akt signaling in PFC, hippocampus and Ml; NR1 levels in these regions positively correlated with MWM performance
Yang et al., 2014	Rat/male/ 2 months/ dementia (VaD, two-vessel occlusion)	*N* = 36/ Control (*n* = 1 0), VaD (*n* = 10), VaD + 1 Hz rTMS (*n* = 8), VaD + 5 Hz rTMS (*n* = 8)	None/restrained manually during stimulation	Spatial memory/ MWM	HE (hippocampal CA1 neuronal morphology); IHC (mTOR and eIF-4E); WB (mTOR and eIF-4E)	rTMS promotes recovery of learning and memory (reduced escape latency and swimming distance, and increased time and distance in target quadrant), both with 1 Hz and 5 Hz frequencies	rTMS restored CA1 neuronal morphology and up-regulated mTOR and eIF-4E protein expression in CA1. 1 Hz and 5 Hz stimulation produced comparable molecular effects
Yang et al., 2015	Rat/male/2 months/ dementia (VaD, two-vessel occlusion)	*N* = 31/control (*n* = 12), VaD (*n* = 10), VaD + rTMS (*n* = 9)	None/restrained manually during stimulation	Spatial memory/ MWM	Electron microscopy (ultrastructure); *in vivo* LTP (synapses); WB (NR1, Bcl- 2 and Bax)	rTMS improved spatial learning and memory (reduced escape latency and distance, and increased target quadrant time)	rTMS attenuated synaptic ultrastructural damage in hippocampal CA1, enhanced CA3-CA1 LTP, up-regulated NR1 and Bcl- 2, and down-regulated Bax expression
Zhang et al., 2015	Rat/male/ /sdults/ dementia (VaD, two- vessel occlusion)	N = 24/control (*n* = 6), VaD (*n* = 6), rTMS (*n* = 6)	None/restrained manually during stimulation	Spatial memory/ MWM	Hippocampal LTP; WB [VEGF, BDNF and NMD A receptors (NR1, NR2A, and NR2B)]	rTMS promotes recovery of learning and memory abilities of VaD rats (reduced escape latency and swimming distance, and increased time and distance in target quadrant)	rTMS enhanced long-term potentiation at hippocampal Cz\3-CA1 synapses and up- regulated VEGF expression. rTMS increased previously low BDNF, NR1 and NR2B expression levels, while NR2A expression remained unchanged
Zhang et al., 2024	Mice/male and female/ 4.33 ± 1.59 months/ healthy	*N* = 57/1 mT+sham (*n* = 10), 1 mT + 10- Hz (*n* = 10), 1 mT + 40-Hz (*n* = 10), 10 mT + sham rTMS (*n* = 9), 10 mT + 10-Hz (*n* = 9), 10 mT + 40-Hz (*n* = 9)	NR/NR	Spatial memory/BM	None	Extremely low intensity rTMS at a frequency of 40 Hz impaired spatial memory in both 10 mT and 1 mT conditions, showing stronger negative effects in the 10 mT condition	None
Zheng et al., 2024	Rat/male/ adult (200–220 g)/ Sleep deprivation (CRSD)	*N* = 60/control (*n* = 15), SD (*n* = 15), SD + sham (*n* = 15), SD + rTMS (*n* = 15)	None/restrained manually during stimulation	Spatial memory/ MWM	Golgi staining (dendritic spine density); TEM (synaptic ultrastructure); RNA-seq; qPCR (KMO); WB (KMO); IF (KMO)	rTMS attenuated spatial memory deficits of SD (reduced escape latency and increased platform crossing and target quadrant time), with no differences observed between the rTMS-treated and control groups	rTMS ameliorated synaptic and ultrastructural alterations induced by SD, including increased dendritic spine density and synapse number. Transcriptomic analysis identified 56 differentially expressed genes. KMO expression was upregulated in SD rats and normalized by rTMS
Zorzo et al., 2021	Rat/male/12 weeks/ healthy	*N* = 20/control (*n* = 10), rTMS (*n* = 10)	None/restrained manually during stimulation	Spatial memory/MWM	Quantitative CCO histochemistry	rTMS not alter spatial learning or memory performance	rTMS reduced CCO activity of the limbic system related to the spatial task

3xTg, Alzheimer’s disease model mice; Aβ, amyloid beta peptide; Aβ_1–42_, amyloid beta peptide of 42 amino acids; AC1, adenylyl cyclase 1; AD, Alzheimer’s disease; Akt, protein kinase B; APP, amyloid precursor protein; APP23/PS45, double transgenic mouse model of Alzheimer’s disease; AQP4, aquaporin-4; ASC, apoptosis associated speck-like protein containing a caspase recruitment domain; BACE1, beta-site APP-cleaving enzyme 1; Bax, Bcl-2–associated X protein; Bcl-2, B-cell lymphoma 2; Bcl-xL, B-cell lymphoma–extra large; BDNF, brain-derived neurotrophic factor; BK, large-conductance calcium-activated potassium channels; BM, barnes maze; BrdU, 5-Bromo-2’-deoxyuridine; C89, 89-amino-acid C-terminal fragment of APP; C99, 99-amino-acid C-terminal fragment of APP; CA1, hippocampal subfield CA1; CA3, hippocampal subfield CA3; cAMP, cyclic adenosine monophosphate; CaMKII, Ca^2+^/calmodulin-dependent protein kinase II; Caspase-1, cysteine-aspartic protease-1; Caspase-3, cysteine-aspartic protease-3; CCO, cytochrome c oxidase; CRSD, chronic REM sleep deprivation; CSF, cerebrospinal fluid; CSF–ISF, cerebrospinal fluid interstitial fluid; cTBS, continuous theta burst stimulation; CUMS, chronic Unpredictable Mild Stress; DAI, diffuse axonal injury; DCX, doublecortin; DEP, depotentiation; DG, dentate gyrus; DMN, default mode network; DMSO, dimethyl sulfoxide; eIF-4E, eukaryotic initiation factor 4E; ELISA, Enzyme-Linked Immunosorbent Assay; FITC, fluorescein isothiocyanate; FJB, Fluoro-Jade B staining; GAP-43, growth-associated protein 43; GAPDH, glyceraldehyde-3-phosphate dehydrogenase; GFAP, glial fibrillary acidic protein; GSDMD, gasdermin D; GSDMD-N, N-terminal fragment of Gasdermin D; HE, hematoxylin and eosin staining; Homer1a/c, isoforms of postsynaptic scaffold protein Homer1 involved in synaptic plasticity; HSP70, heat shock protein 70; Hz, Hertz; Iba1, ionized calcium-binding adapter molecule 1; IF, immunofluorescence; IHC, immunohistochemistry; IL-1β, interleukin-1 beta; K252a, pharmacological inhibitor of Trk receptor; Ki67, antigen identified by monoclonal antibody Ki-67; KMO, kynurenine 3-monooxygenase; LTP, long-term potentiation; LV, lentiviral vector; M1, primary motor cortex; M2, secondary motor cortex; MAP2, microtubule-associated protein 2; MBP, myelin basic protein; MCAO/R, middle cerebral artery occlusion/reperfusion; mRNA, messenger ribonucleic acid; mT, millitesla; MT, motor threshold; mTOR, mammalian target of rapamycin; MWM, Morris Water Maze; NeuN, neuronal nuclei antigen; NF-200, neurofilament 200; NF-κB, nuclear factor kappa B; NGF, nerve growth factor; NLRP3, NOD-like receptor family pyrin domain-containing 3; NMDA, N-methyl-D-aspartate; NR, not reported; NR1, N-methyl-D-aspartate receptor subunit 1; NR2A, N-methyl-D-aspartate receptor subunit 2A; NR2B, N-methyl-D-aspartate receptor subunit 2B; NT 4/5, neurotrophin-4/5; p-Akt, phosphorylated protein kinase B; p-CREB, phosphorylated cAMP response element-binding protein; p-CREB/CREB, phosphorylated cAMP response element-binding protein / total CREB; PFC, prefrontal cortex; PKAc, protein kinase A catalytic subunit; PNS, prenatal restraint stress; PP1, protein phosphatase 1; PPF, paired-pulse facilitation; PSD, postsynaptic density; PSD95, postsynaptic density protein 95; p-TrkB/TrkB, phosphorylated tropomyosin receptor kinase B / total TrkB; qPCR, quantitative polymerase chain reaction; qRT-PCR, quantitative real-time polymerase chain reaction; ReHo, regional homogeneity; RIBI, radiation-induced brain injury; RMT, resting motor threshold; RNA-seq, RNA-sequencing; rs-fMRI, resting-state functional magnetic resonance imaging; rTMS, repetitive transcranial magnetic stimulation; RT-PCR, reverse transcription polymerase chain reaction; SAMP8, senescence-accelerated mouse prone 8; SARM1, sterile alpha and TIR motif-containing 1; SD, sleep deprivation; STP, short-term potentiation; SYN, synaptophysin; TCC, 5-triphenyltetrazolium chloride staining; TEM, transmission electron microscopy; TIMP1, tissue inhibitor of metalloproteinase-1; tMCAO, transient middle cerebral artery occlusion (post-stroke cognitive impairment); TMS, transcranial magnetic stimulation; TNF-α, tumor necrosis factor alpha; TrkB, tropomyosin receptor kinase B; TUNEL, terminal deoxynucleotidyl transferase–mediated dUTP nick end labeling; VaD, vascular dementia; VEGF, vascular endothelial growth factor; WB, western blot; WT, wild-type.

**TABLE 3:** Characteristics and main outcomes of human studies examining the effects of TMS on spatial memory.

References	Sample characteristics: condition/sex/age	Total sample size/groups (*n*)	Study design	Memory domain/task	Neurophysiological measures	Memory outcomes	Neurophysiological outcomes
Asl et al., 2024	Treatmentresistant MDD/men and women/ 18–53 years	*N* = 47/sham (*n* = 17), unilateral rTMS (*n* = 15), bilateral rTMS (*n* = 15)	Between- subject	Visuospatial short-term memory/ Corsi Block-Tapping Test	None	Unilateral rTMS improved visuospatial memory, whereas bilateral rTMS did not produce effects	None
Demirtas-Tatlidede et al., 2010	Schizophrenia/men and women/ 29–54 years	*N* = 8/ iTBS (*n* = 8)	Within- subject	Visuospatial short-term memory/spatial span of WMS- III and ROCF	None	iTBS improved visuospatial memory poststimulation and at follow-up	None
Deng et al., 2024	Healthy/men and women/18–30 years	*N* = 46/sham rTMS (*n* = 23), Real rTMS (*n* = 23); 6 excluded in the EEG analysis (three excluded per group)	Within and between- subjects	Visuospatial short-term memory/spatial span task	EEG	rTMS improved visuospatial shortterm memory’ during stimulation (online spatial span task) and post-stimulation (offline spatial span task)	rTMS increased parietal alpha power during spatial memory maintenance and enhanced contralateral delay activity amplitude at post-stimulation
Dordevic et al., 2022	Healthy/men and women/18–29 years	*N* = 20/sham (NR) vs. cTBS (NR)	Within- subject	Egocentric spatial memory/spatial updating paradigm (blind walking)	None	cTBS impaired egocentric spatial memory during dynamic spatial updating (increased distance and angular errors), with no effects in the static control condition	None
Goldsworthy et al., 2020	Healthy/men and women/young: 18–29 years, older: 52–83	*N* = 66/young (*n* = 33, 13 selected for EEG), Older (*n* = 33, 10 selected for EEG); Sham (NR) vs. iTBS (NR)	Within- subject	Visuospatial associative memory/ PAL and CANTAB	EEG	iTBS not improve visuospatial memory, although PAL performance in older adults was associated with iTBS- induced cortical plasticity	iTBS increased prefrontal cortical excitability (P60 TEP amplitude) in young adults, but not in older adults
Guidali et al., 2019	Healthy/men and women/ 23.5 ± 4.0 years, control: 21.6 ± 2.1 years	N = 35/control (*n* = 15), exp. 2 (*n* = 20)	Within- subject	Visuospatial short-term memory/ Computerized Corsi Block-Tapping Test	None	rTMS over the left SMG selectively impaired visuospatial memory, increasing serial order errors without affecting item errors. rTMS over the IFG not affect performance	None
Hermiller et al., 2022	Healthy/men and women/ young: 21–28 years, Older: 60–73 years	*N* = 30/ young (*n* = 15), older (*n* = 15), cTBS (NR) vs. 20 Hz rIMS (NR)	Within and between- subjects	Visuospatial associative memory/object-location spatial memory task	fMRI (resting-state functional connectivity of the hippocampal network)	cTBS increased swap errors in the visuospatial object-location memory task in young adults, whereas 20 Hz rTMS not produce behavioral changes. No memory effects were observed in older adults	cTBS increased hippocampal network functional connectivity in young adults, whereas 20 Hz rTMS not. In older adults, TBS induced widespread, non-specific connectivity changes
Huang et al., 2024	Healthy/men and women/18–30 years	*N* = 24/sham (NR) vs. active: left, right, and bilateral (NR)	Within- subject	Visuospatial short-term memory/spatial grid memory task	None	cTBS applied bilaterally improved spatial memory compared with sham, with additional prepost improvements after bilateral, right and left application	None
Marin et al., 2018	Healthy/men and women/18–38 years	*N* = 28/sham (*n* = 14), active- TBS(*n* = 14)	Between -subject	Episodic memory with spatial component/ Object-location associative task with active retrieval vs. passive restudy	None	TBS selectively impaired longterm associative recognition and long-term spatial recall in the active-retrieval condition, with no effects on shortterm spatial memory and no effects in the passive-restudy condition	None
Nilakantan et al., 2017	Healthy/men and women/19–35 years	*N* = 16 (*n* = 11 female). additional zerointensity control group: *N* = 12 (*n* = 8 female)	Within and between- subjects	Episodic visuospatial memory/object-location memory task	EEG and fMRI	rTMS improved memory precision, without affecting general recollection success. No memory improvement was observed in the sham or zerointensity control conditions	rTMS reduced theta-alpha oscillatory activity and late positive ERP amplitudes during recollection, and these neural changes were associated with the improvement in memory precision
Tang et al., 2023	Clinical high-risk for psychosis and first-episode schizophrenia/men and women/15–45 years	*N* = 58/ sham rIMS (*n* = 27), rIMS (*n* = 31)	Between -subject	Visuospatial learning and memory/BVMT-R	None	TMS improved visuospatial learning (higher BVMT-R total scores and increased individual visuospatial learning precision). Effects were also replicated in an independent first- episode schizophrenia sample	None
Traikapi et al., 2023	Probable Alzheimer’s disease/men and women/70.75 ± 5.06 years	*N* = 4	Within- subject	Visuospatial short-term memory/ Corsi Block-Tapping Task	None	rTMS not lead to any improvement in visuospatial short-term memory	None

BVMT-R, brief visuospatial memory test–revised; CANTAB, computer-based cambridge neuropsychological test automated battery; cTBS, continuous theta burst stimulation; EEG, electroencephalography; ERP, event-related potential; fMRI, functional magnetic resonance imaging; IFG, inferior frontal gyrus; iTBS, intermittent theta burst stimulation; MDD, major depressive disorder; NR, not reported; PAL, paired associates learning; ROCF, rey osterrieth complex figure; rTMS, repetitive transcranial magnetic stimulation; SMG, supramarginal gyrus (inferior parietal lobule); TBS, theta burst stimulation; TEP, transcranial magnetic stimulation-evoked cortical potential; WMS-III, Wechsler Memory Scale–Third Edition.

**TABLE 4:** Stimulation parameters of rodent studies examining the effects of TMS on spatial memory.

References	Protocol type	Coil type	Stimulation area	Motor threshold (mt) determination	Intensity/frequency	Number of pulses per session	Train: number/ duration/ inter-train interval	Sessions: number per day/ intersession interval/ daily duration/ total duration
Bao et al., 2021	rTMS	NR	Top of the skull	NR	1.2 T/5 Hz	1,000	10/20 s/20 s	1/NR/6 min 50 s/14 consecutive days
Hong et al., 2024	rTMS	Round coil (customized magnetic stimulator, YRD-CCI, Wuhan, China)	Parietal cortex (over ischemic hemisphere)	Minimum stimulation intensity eliciting visible motor twitches in the non-paralyzed limb (MT- based)	MT + 10%/20 Hz	2,000	10/10 s/50 s	1/NR/10 min/21 consecutive days
Huang et al., 2017	rTMS	Round coil (CCY-III, Wuhan Yiruide Medical Equipment Co., LTD., Wuhan, China)	Centered over the exposed head	100% average RMT determined by visible bilateral forelimb movement in anesthetized mice (visual inspection-based)	100% RMT/1 Hz	600	20/30 s/2 s	1/NR/10 min 40 s/14 consecutive days
Jiang et al., 2023	rTMS	Circular coil (CCY-I magnetic stimulator)	Over the hemisphere	NR	100% of the stimulator output/10 Hz	6,000	NR/NR/10 s	1/NR/10 min/15 days (three blocks of 5 days with 2-day intervals, 3 weeks total)
Jiang et al., 2025	TMS	Circular Coil (Ml00, Shenzhen Yingchi Technology Co., Ltd, China)	Top of the skull	120% RMT determined of minimum stimulus intensity the MEPs amplitude in the quadriceps femoris muscle of the left hind limb was greater than 15 μV in at least 5 out of 10 consecutive stimuli	120% RMT/10 Hz	300	10/3 s/50 s	1/NR/8 min 50 s/7 consecutive days
Kim et al., 2024	rTMS	Figure-8-shaped coil (Magstim Rapid2, MagStimR, Whitland, UK)	Over the frontal cortex	NR	50% RMT/1 Hz	100	10/ 10 s/50 s	1/NR/10 min/5 consecutive days
Li et al., 2007	rTMS	Round coil (Biomedical Engineering Unit, China)	Top of the skull	NR	I.O.T/0.5 Hz	300	6/~100 s/30 s	1/NR/13 min/ chronic: 10 consecutive days; acute: 1 day
Lin et al., 2024	cTBS	Figure-8-shaped coil (Yiruide CCY- IA magnetic stimulation)	Top of the skull (above cranial windows)	NR	20% maximum output/bursts of three pulses at 50 Hz repeated at 5 Hz	600	1/40 s/NR	1/NR/40 s/14 consecutive days
Ma et al., 2014	rTMS	Butterfly coil (MC-B70 MagPro X100, Mag Venture, Denmark)	Top of the skull	Average RMT determined by visual inspection of bilateral forelimb movement under anesthesia	110% and 150% RMT/1 Hz	400	5/20 s/10 s	4/30 s/∼10 min/14 consecutive days
Ma et al., 2017	rTMS	Butterfly coil (MC-B70 MagPro X100, MagV enture, Denmark)	Top of the skull	NR	30% maximum output (1.26 T)/5 Hz	500	10/10 s/20 s	2/ 2 min/5 min/14 consecutive days
Ma et al., 2019	rTMS	Butterfly coil (MC-B70 MagPro X100, Mag Venture, Denmark)	Top of the skull	NR	20% maximum output (0.84 T)/5 or 25 Hz	1,000	5 Hz: 10/20 s/30 s; 25 Hz: 10/4 s/30 s	1/NR/5 Hz: 8 min, 20 s; 25 Hz: 5 min, 40 s/14 consecutive days
**Qin et al., 2024**	rTMS	Round prototype coil	Top of the skull	NR	NR/10 Hz	500	NR/NR/NR	2/2 min/ NR/4 days
Shang et al., 2016	rTMS	Figure-8-shaped coil (Rapid2 Magstim, UK)	Parietal cortex	AMT determined by visual inspection of bilateral forelimb movement	120% MT (28% of maximum output)/5 Hz	400	20/4 s/20 s	1/NR/8 min/14 consecutive days
Shang et al. (2019)	rTMS	Figure-8-shaped coil	Top of the skull	NR	NR/5 Hz	400	20/4 s/20 s	1/NR/8 min/14 days
Tan et al., 2013	rTMS	Round coil (high-Speed MES-10, Cadwell, USA)	Top of the skull	AMT determined by visual inspection of bilateral forelimb movement	100% RMT (20% maximum output, 0.4 T)/1 Hz	400	20/20 s/10 s	1/ NR/ 10 min/14 consecutive days
Wang et al., 2015	rTMS	Figure-8-shaped coil for rodents (Magstim Rapid, MRS1000/50, Magstim Company Ltd, Whitland, UK)	Over the skull	NR	80% of the maximum output/1, 10, and 15 Hz	1 Hz: 5; 10 Hz: 50; 15 Hz: 75	1/5 s/NR	1/-/5 s/20 days (4 weeks, weekdays)
Wu et al., 2022	rTMS	Circular coil (Y064, rodentspecific, Wuhan Yiruide Medical Equipment Co., LTD, China)	PFC; coil center over the middle of the interocular line	Mean MT = 42.60 ± 9.10% of maximum stimulator output	80% average MT/10 Hz	1,000	10/10 s/NR	1/NR/10 min/20 days (4 weeks, weekdays)
Yang et al., 2014	rTMS	Figure-8-shaped coil (MC-B70, Medtronic, Denmark)	Over the rat sagittal suture central point, close to the scalp surface and parallel to the parietal bone	NR	Low frequency: 0.5 T/1 Hz; high frequency: 0.5 T/5 Hz	600	1 Hz: 1/600 s/NR; 5 Hz: 30/4 s/2 s	1 Hz: 1/NR/ 10 min/ 10 days (2 weeks, weekday); 5 Hz: 1/NR/2 min 58 s/10 days (2 weeks, weekday)
Yang et al., 2015	rTMS	Figure-8-shaped coil (MC-B70, Medtronic, Denmark)	Over the rat sagittal suture central point, close to the scalp surface and parallel to the parietal bone	NR	0.5 T/1 Hz	600	1/600 s/NR	1/ NR/10 min/ 10 days(2 weeks, weekday)
Zhang et al., 2015	rTMS	Figure-8-shaped coil (MC-B70, Tianjin, China)	Over the rat sagittal suture central point close to the scalp surface and parallel to the parietal bone.	NR	120% of the average MT (0.5 T)/ 5 Hz	200	20/2 s/2 s	1/NR/1 min 18 s/20 days (4 weeks, weekdays)
Zhang et al., 2024	rTMS	Round coil; diameter: 25 mm; height: 11 mm (Tianjin Langshuo Electric Co. LTD, Tianjin, China)	Over the skull	NR	10 mT and 1 mT/ 40 Hz and 10 Hz	10 Hz: 18,000; 40 Hz: 72,000	NR/continuous/ NR	1/NR/ 30 min/11 consecutive days
Zheng et al. (2024)	rTMS	Circular prototype coil (NTK- TMS-II, Jiangxi, China)	Over the skull	Lowest stimulation intensity eliciting visible bilateral lower- extremity twitching (visual inspectionbased)	80% of the average MT (3 T)/ 1 Hz	50	1/ 20 s/ NR	l/NR/25 s/ 14 consecutive days
Zorzo et al., 2021)	rTMS	Small magnetic head made of nanocrystallin e material (Vitroperm 500F)	Over the skull, at the height of the retrosplenial cortex	NR	0.33 T/ 100 Hz	30000	NR/NR/ 30s	1/ NR/10 min/ 3 consecutive days

AMT, active motor threshold; cTBS, continuous theta burst stimulation; DLPFC, dorsolateral prefrontal cortex; EMG, electromyography; Hz, hertz; IPL, inferior parietal lobule; MEP, motor evoked potential; Min, minutes; MT, motor threshold; NR, not reported; PFC, prefrontal cortex; RMT, resting motor threshold; rTMS, repetitive transcranial magnetic stimulation; T, tesla; TMS, transcranial magnetic stimulation; UK, United Kingdom; USA, United States of America; μV, microvolts.

**TABLE 5:** Stimulation parameters of human studies examining the effects of TMS on spatial memory.

References	Protocol type	Coil type	Stimulation area	Motor threshold (MT) determination	Intensity/ frequency	Number of pulses per session	Train: number/duration/ inter-train interval	Sessions:number per day/inter-session interval/ daily duration/total duration
Asl et al. (2024)	rTMS	Figure-of- eight coil (Magstim super rapid, UK)	Left DLPFC (unilateral), left DLPFC + right DLPFC (bilateral)	Minimum TMS intensity evoking > 50 μV MEP in right APB in >5/10 trials (EMG-based)	Unilateral: 85% RMT/ 20 Hz; Bilateral: 85% RMT/Left 20 Hz/and 110% RMT/ Right 1 Hz	Unilateral: 2200; Bilateral: 1400 left + 800 right (2,200 total)	NR/NR/NR	1/NR/NR/10 days (2 weeks, weekdays)
Demirtas-Tatlidede et al., 2010	iTBS	Figure-of- eight coil (Tonica, Denmark)	Cerebellar vermis	Individual AMT	100% AMT/bursts of three pulses at 50 Hz repeated at 5 Hz	600	20/NR/8 s	2/∼5 h/NR/5 consecutive days
Deng et al., 2024	rTMS	Figure-of- eight coil (MagStim, Whitland, UK)	Parietal cortex	Minimum TMS intensity evoking > 50 μV MEP in first dorsal interosseus muscle > 5/10 trials	100% RMT/10 Hz	600	NR/NR/NR	2/30 min/ NR/3 consecutive days
Dordevic et al., 2022	cTBS	Figure-of- eight coil (Cool B-65, Mag Venture, Denmark)	Right posterior parietal cortex (right precuneus)	Minimum intensity evoking > 100 μV MEP in right APB in >5/10 trials (EMG-based)	100% RMT/bursts of three pulses at 30 Hz delivered at 6 Hz	801	1/44 s/NR	1/NR/44 s/1 day
Goldsworthy et al., 2020	iTBS	Figure-of- eight coil (Magstim, Whitland, UK)	Left DLPFC	Minimum TMS intensity evoking > 50 μV MEP in right first dorsal interosseous in >5/10 trials (EMG-based)	70% RMT/bursts of three pulses at 50 Hz repeated at 5 Hz	600	16/2 s/ 10 s	1/NR/3 min 12 s/1 day
Guidali et al., 2019	rTMS	Figure-of- eight coil (Magstim, Whitland, UK)	Left SMG, Left IFG	Minimum stimulator output eliciting a detectable motor twitch in the right hand in >5/10 trials during left Ml stimulation (RMT-based)	100% RMT/1 Hz	600	1 continuous/10 min/NR	1/NR/10 min/1 day
Hermiller et al., 2022	rTMS and cTBS	Butterfly coil (MagV enture A/S, Denmark)	Parietal cortex	Minimum stimulator output eliciting a detectable motor response in the right APB in >5/10 trials (RMT-based)	rTMS: 80% RMT/20 Hz; cTBS: 80% RMT/bursts of three pulses at 50 Hz repeated at 5 Hz	rTMS: 600; cTBS: 600	rTMS: 15/2 s/28 s; cTBS: continuous stimulation/40 s/NR	rTMS: 1/NR/7 min 3 s/1 day; cTBS: l/NR/40 s/1 day
Huang et al., 2024	cTBS	Figure-eight coil (Magneuro 100, Vishee Medical Technology Co., Ltd., Nanjing, China)	Cerebellum Crus I region	Minimum TMS intensity evoking > 200 μV MEP in >5/10 trials (MRI-based)	80% of the AMT/ bursts of three pulses at 50 Hz repeated at 5 Hz	600	Continuous stimulation/40 s/NR	1/NR/40 s/1 day
Marin et al., 2018	cTBS	Figure-eight coil (Nexstim eXimia NBS 4.3, Nexstim Ltd.)	Right DLPFC	Minimum intensity evoking > 50 μV MEP in abductor pollicis brevis in >6/10 trials (surface EMG)	Sham: 20% MT/ active: 80% MT/5 Hz	600	Continuous stimulation/40 s/NR	1/NR/40 s/1 day
Nilakantan et al., 2017	rTMS	Figure-of- eight coil (Nexstim eXimia NBS 4.3, Nexstim Ltd)	Hippocampal posterior- medial network	Minimum stimulator output required to generate a contraction of the abductor pollicis brevis for five consecutive pulses, measured visually or via EMG (50 μV)	100% MT/20 Hz	1,600	40/2 s/28 s	1/NR/20 min/5 consecutive days
Tang et al., 2023	rTMS (personali zed network - based)	Standard round coil (MCF-B65 coil), 9 cm in diameter (MagPro X100 stimulator, Medtronic, Denmark)	Left IPL	MT defined as the lowest TMS intensity evoking a > 50 **μ**V MEP in the right abductor pollicis brevis muscle	110% RMT/20 Hz	1,500	NR/NR/NR	5/60 min/NR/2 consecutive days
Traikapi et al., 2023	rTMS	Figure-of- eight coil (Magstim Super Rapid Plus, UK)	Left and right precuneus (bilateral, alternating days)	Minimum intensity eliciting MEPs > 50 pV in >5/10 trials in the first dorsal interosseous muscle (EMG- based)	~80%–90% RMT/40 Hz	1,000	25/1 s/29 s	1/NR/12 min 30 s/10 days (2 weeks, weekdays)

AMT, active motor threshold; APB, abductor pollicis brevis; cTBS, continuous theta burst stimulation; DLPFC, dorsolateral prefrontal cortex; EMG, electromyography; h, hour; IFG, inferior frontal gyrus; IPL, inferior parietal lobule; iTBS, intermittent theta burst stimulation; MEP, motor evoked potential; MRI, magnetic resonance imaging; NR, not reported; PFC, prefrontal cortex; RMT, resting motor threshold; rTMS, repetitive transcranial magnetic stimulation; SMG, supramarginal gyrus; TMS, transcranial magnetic stimulation; UK, United Kingdom; μV, microvolts.

### 3.1 Sample characteristics and experimental designs

#### 3.1.1 Animal studies

This section summarizes the main characteristics of the included animal studies, focusing on experimental models, sample (species, sex and age) and study design. Detailed study- level information is provided in Table 2.

Both healthy cohorts and pathological models were represented. Several studies included healthy or normally aged animals (7; 30.4%). Pathological models included Alzheimer’s disease, vascular dementia, stroke, brain injury, stress- and depression-related paradigms, sleep deprivation, and accelerated aging models, whereas seven studies were conducted in healthy animals.

Among the included animal studies, nine studies (39.1%) were conducted in mice (Wang et al., 2015; Huang et al., 2017; Ma et al., 2017, 2019; Bao et al., 2021; Lin et al., 2024; Qin et al., 2024; Zhang et al., 2024) and 14 (60.9%) in rats (Li et al., 2007; Tan et al., 2013; Yang et al., 2015; Zhang et al., 2015; Shang et al., 2016, 2019; Zorzo et al., 2021; Jiang et al., 2023, 2025; Hong et al., 2024; Zheng et al., 2024; Kim et al., 2024). Regarding sex, most studies (22; 95.7%) used male-only samples, while only one study (4.3%) included both sexes (Zhang et al., 2024). No study employed female-only cohorts. Most studies used young-adult animals (73.9%), whereas adult samples without precise age reporting accounted for 21.7%. Only two studies employed aged animals and one did not report age (see Table 2).

Most studies were conducted in adult animals, with ages ranging from approximately 7 weeks to 15 months. Regarding experimental design, 95.6% of studies included a baseline comparison condition (healthy controls or untreated pathological models), with only one study lacking either a healthy or sham-stimulation comparison group. Sham-stimulation groups were reported in 60.9% of studies, whereas 56.5% included control groups, and 21.7% incorporated both conditions. At the group level, sample sizes were typically ranging between *n* = 6 and *n* = 15 animals per group, although larger group sizes were also reported in some studies [e.g., *n* = 20–25 per group in Bao et al. (2021), Jiang et al. (2025)].

#### 3.1.2 Human studies

This section summarizes the main characteristics of the included human studies, focusing on participant characteristics (clinical vs. non-clinical status, sex/gender, and age) and study design. Detailed study-level information is provided in Table 3.

The 12 human studies included in this review comprised samples of both clinical and non-clinical adult populations. Most studies recruited healthy participants (8; 66.7%) (Nilakantan et al., 2017; Marin et al., 2018; Guidali et al., 2019; Goldsworthy et al., 2020; Dordevic et al., 2022; Hermiller et al., 2022; Deng et al., 2024; Huang et al., 2024). Clinical cohorts were less represented, with one study in each condition (8.3%) focusing on treatment-resistant major depressive disorder (Asl et al., 2024), schizophrenia (Demirtas-Tatlidede et al., 2010), psychosis-spectrum populations, including clinical high-risk and first-episode schizophrenia (Tang et al., 2023), and probable Alzheimer’s disease (Traikapi et al., 2023).

Regarding sex/gender distribution, all studies included both men and women. Participant ages ranged from adolescence to older adulthood, although young adults predominated (50.0%). Two studies included broader adult age ranges, two directly compared younger and older adults, and the remaining clinical studies reflected the age profiles of the corresponding disorders, including psychosis-spectrum populations and probable Alzheimer’s disease (see Table 3).

### 3.2 Stimulation protocols and parameters

#### 3.2.1 Animal studies

This section summarizes the main stimulation parameters, focusing on TMS protocol (rTMS or TBS), stimulation session structure (pulses delivered, train organization, and session scheduling), frequency and intensity, brain target area, coil, and animal handling during stimulation. Detailed study-level information is provided in Table 4.

Most preclinical protocols employed rTMS (95.6%), whereas only one study (Lin et al., 2024) used cTBS (4.3%).

Stimulation protocols across animal experiments varied widely in pulse count (5 to 72,000 per session) (Wang et al., 2015; Zhang et al., 2024), treatment duration (1–21 days) (Li et al., 2007; Hong et al., 2024), and stimulation frequency (0.5–100 Hz) (Li et al., 2007; Hong et al., 2024), making cross-study comparisons largely descriptive rather than inferential. The number of TMS pulses delivered per session fell into four ranges: ≤400 pulses used in 10 of 23 studies (43.5%) (Li et al., 2007; Tan et al., 2013; Wang et al., 2015; Zhang et al., 2015; Shang et al., 2016, 2019; Kim et al., 2024; Zheng et al., 2024; Jiang et al., 2025); between 401 and 1,000 pulses in nine studies (39.1%) (Yang et al., 2014, 2015; Wang et al., 2015; Huang et al., 2017; Ma et al., 2017, 2019; Bao et al., 2021; Wu et al., 2022; Lin et al., 2024; Qin et al., 2024); between 1,001 and 2,000 in one study (4.3%) (Hong et al., 2024) and three studies (13.0%) reported > 2,000 pulses per session (Zorzo et al., 2021; Jiang et al., 2023; Zhang et al., 2024). Regarding the sessions performed, most studies administered one TMS session per day (20; 87.0%) (Li et al., 2007; Tan et al., 2013; Yang et al., 2014, 2015; Wang et al., 2015; Zhang et al., 2015, 2024; Shang et al., 2019, 2016; Huang et al., 2017; Ma et al., 2019; Bao et al., 2021; Zorzo et al., 2021; Wu et al., 2022; Jiang et al., 2023, 2025; Hong et al., 2024; Zheng et al., 2024; Kim et al., 2024; Lin et al., 2024). Two sessions per day were administered in two studies (8.7%) (Ma et al., 2017; Qin et al., 2024), and four sessions were administered in the remaining study (4.3%) (Ma et al., 2014). For a more detailed information about train organization and inter session intervals, see Table 4. The total treatment duration most often lasted 14 days (10; 43.5%) (Tan et al., 2013; Ma et al., 2014, 2017, 2019; Shang et al., 2016, 2019; Huang et al., 2017; Bao et al., 2021; Lin et al., 2024; Zheng et al., 2024), followed by 20 days (3; 13.0%) (Wang et al., 2015; Zhang et al., 2015; Wu et al., 2022) and 10 days (13.0%) (Yang et al., 2014, 2015; Zhang et al., 2015). Other treatment durations were less frequently reported and included 21 days (Hong et al., 2024), 15 days (Jiang et al., 2023), 11 days (Zhang et al., 2024), 7 days (Jiang et al., 2025), 5 days (Kim et al., 2024), 4 days (Qin et al., 2024), 3 days (Zorzo et al., 2021), and 1 day (Li et al., 2007), each representing a single study (4.3%).

Regarding stimulation frequency, seven of the 23 studies (30.4%) used low-frequency protocols (≤1 Hz) (Li et al., 2007; Tan et al., 2013; Ma et al., 2014; Yang et al., 2015; Huang et al., 2017; Kim et al., 2024; Zheng et al., 2024), while 14 studies (60.9%) applied high-frequency protocols (≥5 Hz) (Zhang et al., 2015, 2024; Shang et al., 2016, 2019; Ma et al., 2017, 2019; Bao et al., 2021; Zorzo et al., 2021; Wu et al., 2022; Jiang et al., 2023, 2025; Hong et al., 2024; Lin et al., 2024; Qin et al., 2024). Mixed low and high frequencies schedules were implemented in two studies (8.7%) (Yang et al., 2014; Wang et al., 2015), and TBS protocol consisted of bursts of three pulses at 50 Hz repeated at 5 Hz in one (4.3%) (Lin et al., 2024).

With respect to the brain target area where the stimulation coil was positioned, most preclinical studies applied broadly distributed cranial stimulation rather than focal cortical targeting. In 18 of the 23 studies (78.3%), the coil was placed over the skull or along the dorsal midline, using descriptions such as “top of the skull,” “over the skull,” mid-sagittal positioning, or centering over cranial windows or exposed skull (Li et al., 2007; Tan et al., 2013; Yang et al., 2014, 2015; Ma et al., 2014, 2017, 2019; Wang et al., 2015; Zhang et al., 2015, 2024; Huang et al., 2017; Shang et al., 2019; Bao et al., 2021; Jiang et al., 2023, 2025; Zheng et al., 2024; Lin et al., 2024; Qin et al., 2024). In contrast, five of the 23 studies (21.7%) reported focal cortical targeting: the parietal cortex (Shang et al., 2016), the frontal cortex (Kim et al., 2024), the prefrontal cortex (Wu et al., 2022), and the retrosplenial cortex (Zorzo et al., 2021). Consistent with these differences in targeting strategy, the type of the stimulation coil was also variable. Approximately half of the studies (11; 47.8%) used figure-of-eight (butterfly) coils (Ma et al., 2014, 2017, 2019; Yang et al., 2014, 2015; Zhang et al., 2015; Wang et al., 2015; Shang et al., 2016, 2019; Kim et al., 2024; Lin et al., 2024), while another 10 studies (43.5%) used round/circular coils (Li et al., 2007; Tan et al., 2013; Huang et al., 2017; Zorzo et al., 2021; Wu et al., 2022; Jiang et al., 2023, 2025; Hong et al., 2024; Qin et al., 2024; Zhang et al., 2024), and one study (4.3%) used an ad hoc prototype with a small-head coil (Zorzo et al., 2021). Finally, one study (4.3%) did not report the coil type (Bao et al., 2021).

Regarding animal handling during stimulation, awake restraint was used in the majority of studies (73.9%), while two studies (8.7%) used anesthesia, and the remaining four (17.4%) did not report application mode.

#### 3.2.2 Human studies

This section summarizes the main stimulation parameters, focusing on TMS protocol (rTMS or TBS), stimulation session structure (pulses delivered, train organization, and session scheduling), frequency and intensity, brain target area and coil. Detailed study- level information is provided in Table 5.

In human studies (*n* = 12), rTMS was the most frequently used protocol, appearing in seven studies (58.3%) (Nilakantan et al., 2017; Guidali et al., 2019; Hermiller et al., 2022; Tang et al., 2023; Traikapi et al., 2023; Asl et al., 2024; Deng et al., 2024), while TBS in six (50.0%), including iTBS in two (16.7%) (Demirtas-Tatlidede et al., 2010; Goldsworthy et al., 2020) and cTBS in five (41.7%) (Marin et al., 2018; Dordevic et al., 2022; Hermiller et al., 2022; Huang et al., 2024). To note, one study combined rTMS and cTBS (Hermiller et al., 2022). Regarding structure of stimulation (number of pulses/session, arrangement of the trains, and sessions), the total of the studies can be grouped into four categories: ≤ 600 pulses (7; 58.3%) (Demirtas-Tatlidede et al., 2010; Marin et al., 2018; Guidali et al., 2019; Goldsworthy et al., 2020; Hermiller et al., 2022; Deng et al., 2024; Huang et al., 2024); 601–1,000 pulses (2; 16.7%) (Dordevic et al., 2022; Traikapi et al., 2023), 1,001–2,000 pulses (2; 16.7%) (Nilakantan et al., 2017; Tang et al., 2023), and more than 2,000 pulses in one study (8.3%) (Asl et al., 2024). Both multi-train (41.7%) and single-train/continuous protocols (41.7%) were commonly used, whereas train organization was not specified in 25.0% of studies (for a more detailed information see Table 5).

As for session-level parameters, one session per day was applied in most studies (9; 75.0%) (Nilakantan et al., 2017; Marin et al., 2018; Guidali et al., 2019; Goldsworthy et al., 2020; Dordevic et al., 2022; Hermiller et al., 2022; Traikapi et al., 2023; Asl et al., 2024; Huang et al., 2024), two sessions in two (16.7%) (Demirtas-Tatlidede et al., 2010; Deng et al., 2024) and five sessions in one study (8.3%) (Tang et al., 2023). Daily duration was detailed in eight studies (66.7%) (Nilakantan et al., 2017; Marin et al., 2018; Guidali et al., 2019; Goldsworthy et al., 2020; Dordevic et al., 2022; Hermiller et al., 2022; Traikapi et al., 2023; Huang et al., 2024), and the total intervention duration lasted one day in half of the studies (50.0%) (Marin et al., 2018; Guidali et al., 2019; Goldsworthy et al., 2020; Dordevic et al., 2022; Hermiller et al., 2022; Huang et al., 2024); 5 days in two (16.7%) (Demirtas-Tatlidede et al., 2010; Nilakantan et al., 2017); 10 days in two (16.7%) (Traikapi et al., 2023; Asl et al., 2024); 3 days in one (8.3%) (Deng et al., 2024), and 2 days in one study (8.3%) (Tang et al., 2023).

In terms of stimulation frequency, high-frequency rTMS protocols predominated in the human literature. Specifically, ≥10 Hz was applied in four of the 12 studies (33.3%) (Nilakantan et al., 2017; Hermiller et al., 2022; Tang et al., 2023; Deng et al., 2024), while low-frequency (1 Hz) was less frequently employed, appearing as a primary protocol in one study (8.3%) (Guidali et al., 2019). In addition, one study (8.3%) implemented a combined-frequency bilateral protocol, applying 20 Hz to the left hemisphere and 1 Hz to the right hemisphere within the same intervention (Asl et al., 2024). By contrast, TBS constituted a substantial proportion of the stimulation approaches, being reported also in five studies (41.7%), including iTBS in two studies (16.7%) (Demirtas-Tatlidede et al., 2010; Goldsworthy et al., 2020) and cTBS in three studies (25.0%) (Marin et al., 2018; Dordevic et al., 2022; Huang et al., 2024). Notably, one study (8.3%) directly compared high-frequency rTMS and TBS within the same experimental design (Hermiller et al., 2022).

Brain stimulation targets clustered primarily within frontal, parietal and cerebellar networks. The dorsolateral prefrontal cortex was selected in three studies (25.0%) (Marin et al., 2018; Goldsworthy et al., 2020; Asl et al., 2024). Posterior parietal or adjacent posterior networks constituted the most frequently stimulated regions, being targeted in five studies (41.7%). These included the parietal cortex (Hermiller et al., 2022; Deng et al., 2024), the right (Dordevic et al., 2022) and bilateral precuneus (Traikapi et al., 2023), and the inferior parietal lobule (Tang et al., 2023). Two studies (16.7%) focused on cerebellar regions, specifically the vermis (Demirtas-Tatlidede et al., 2010) and crus I (Huang et al., 2024). Two additional studies (16.7%) targeted associative networks beyond the canonical dorsal fronto-parietal spatial axis, including the left inferior frontal gyrus/supramarginal gyrus (Guidali et al., 2019) and the posterior-medial hippocampal network (Nilakantan et al., 2017). Regarding coil type, stimulation was predominantly delivered using focal coils. Figure-of-eight coils were used in 10 studies (83.3%) (Demirtas-Tatlidede et al., 2010; Nilakantan et al., 2017; Marin et al., 2018; Guidali et al., 2019; Goldsworthy et al., 2020; Dordevic et al., 2022; Traikapi et al., 2023; Asl et al., 2024; Deng et al., 2024; Huang et al., 2024), whereas butterfly coils were used in one study (8.3%) (Hermiller et al., 2022). The remaining study (8.3%) used a standard 9-cm diameter coil (Tang et al., 2023).

### 3.3 Effects on memory outcomes

#### 3.3.1 Animal studies

This section summarizes the effects of TMS on spatial learning and memory outcomes in animal models. Results are organized according to model type (pathological vs. healthy animal models) and direction of cognitive effects, with an emphasis on the most frequently reported performance measures. Detailed information is provided in Table 2.

Across the 23 animal studies, spatial learning and memory were assessed mainly in the Morris Water Maze (MWM), a labyrinth employed in 21 of the 23 studies (95.6%) while the Barnes maze was used in one study (4.3%) (Zhang et al., 2024). Within the MWM, spatial learning during acquisition was most commonly indexed by escape latency during acquisition trials, as well as time spent in the target quadrant and the number of platform crossings during probe trials. One additional paradigm was used to assess short-term spatial memory using the Y-maze (4.3%) (Jiang et al., 2023).

As stated before, the majority of studies were conducted in pathological or experimentally induced injury models. Among the pathological models, Alzheimer’s disease-related models were the most frequently represented (4; 17.4%), and all studies consistently reported that rTMS attenuated spatial learning and memory deficits relative to non-stimulated controls (Tan et al., 2013; Wang et al., 2015; Huang et al., 2017; Bao et al., 2021). These improvements were primarily evidenced by reduced escape latency and increased time spent in the target quadrant in the MWM. Importantly, one study further demonstrated that these beneficial effects were frequency-dependent, with significant improvements observed at 15 Hz but not at 10 Hz or 1 Hz, highlighting the relevance of stimulation parameters in modulating therapeutic efficacy (Wang et al., 2015). Age- related cognitive decline was examined in one study (4.3%) using a senescence model, observing an improvement following rTMS, while the performance in the stimulated SAMP8 mice remained lower than that of age-matched control mice (Ma et al., 2017).

Similarly, vascular dementia models (3; 13.0%), consistently showed that rTMS promotes recovery of learning and memory (Yang et al., 2014, 2015; Zhang et al., 2015). Notably, beneficial effects were observed at both low (1 Hz) and higher (5 Hz) stimulation frequencies (Yang et al., 2014).

Stroke-related models (3; 13.0%) evaluated the effects of TMS in spatial memory and learning utilizing a transient middle cerebral artery occlusion (Hong et al., 2024; Jiang et al., 2025) or microinfarct (Lin et al., 2024). In these cases, stimulation improved spatial learning and memory performance. Similarly, brain injury models (2; 8.7%) also showed improved spatial memory performance following rTMS (Kim et al., 2024; Qin et al., 2024). In a radiation-induced brain injury model, rTMS significantly improved MWM performance; importantly, these effects were abolished by pharmacological blockade of brain-derived neurotrophic factor (BDNF) signaling (Qin et al., 2024). Similarly, in a diffuse axonal injury model, rTMS significantly reduced escape latency compared with non-stimulated injured animals (Kim et al., 2024).

Psychiatric-related models based on stress-induced depression likewise demonstrated cognitive- enhancing effects of rTMS (2; 8.7%) (Shang et al., 2019; Jiang et al., 2023). Jiang et al. (2023) used a Chronic Unpredictable Mild Stress model of depression, where rTMS reversed short-term spatial memory deficits, as evidenced by restored novel arm preference in the Y-maze test (Jiang et al., 2023). On the other hand, Shang et al. (2019), used a Prenatal Restraint Stress model and found that rTMS ameliorated the induced spatial learning and reference memory deficits in rats, as shown by a reduced escape latency and path length, and increased cognitive strategy scores, target quadrant dwell time and platform crossings in the MWM. Furthermore, there was one sleep deprivation model (4.3%) (Zheng et al., 2024). In this study, adult rats were subjected to sleep deprivation and then treated with rTMS, finding that rTMS attenuated spatial learning and memory deficits, with treated animals performing at levels comparable to non-sleep- deprived controls.

A smaller number of studies included healthy animals (7; 30.4%) (Li et al., 2007; Ma et al., 2014, 2019; Shang et al., 2016; Zorzo et al., 2021; Wu et al., 2022; Zhang et al., 2024). Among these studies, five were conducted on healthy rats/mice (21.7%), two of which (2/5; 40.0%) found that rTMS improved spatial learning and memory deficits in healthy adult rats, as evidenced by a reduced escape latency and an increase in time spent in the target quadrant (Shang et al., 2016; Wu et al., 2022); the remaining three (3/5; 60.0%) reported either no significant effects or impaired spatial learning (Li et al., 2007; Zorzo et al., 2021; Zhang et al., 2024). In healthy rats, Li et al. (2007) found that chronic rTMS impaired both short-term and long-term spatial reference memory, whereas acute rTMS only impaired long-term spatial reference memory, without affecting acquisition.

Conversely, extremely low-intensity rTMS applied at 40 Hz in healthy mice significantly impaired spatial memory performance in the Barnes maze, with stronger negative effects observed at 10 mT compared to 1 mT (Zhang et al., 2024). Lastly, Zorzo et al. (2021) reported no significant effects of rTMS on spatial learning or memory performance in healthy 3-month-old rats.

Finally, two studies (8.7%) focused on normally aged animals (Ma et al., 2014, 2019). Ma et al. (2019) found that rTMS attenuated spatial memory impairment in normally aged mice, as shown in a reduction in escape latency and increased platform crossings, with greater improvement at 5 than 25 Hz. Another study conducted by Ma et al. (2014) found an improvement in normally aged mice only when applied at 110% resting motor threshold (RMT), whereas 150% RMT worsened spatial memory performance.

#### 3.3.2 Human studies

This section summarizes the effects of TMS on spatial learning and memory outcomes in human participants. Results are organized according to sample characteristics (clinical vs. healthy populations) and memory domain, with an emphasis on the most frequently reported behavioral measures. Detailed information is provided in Table 3.

Across the 12 human studies, spatial and visuospatial memory was assessed using a range of standardized neuropsychological and experimental tasks. Visuospatial short-term memory was the most frequently assessed domain (6; 50.0%) (Demirtas-Tatlidede et al., 2010; Guidali et al., 2019; Traikapi et al., 2023; Asl et al., 2024; Deng et al., 2024; Huang et al., 2024), primarily measured using the Corsi Block-Tapping Test and Spatial Span tasks. Additional paradigms included a spatial grid memory task (Huang et al., 2024) and the Rey–Osterrieth Complex Figure (Demirtas-Tatlidede et al., 2010). Object–location memory tasks were used in four studies (33.3%) (Nilakantan et al., 2017; Marin et al., 2018; Goldsworthy et al., 2020; Hermiller et al., 2022). One study used a spatial updating paradigm based on blind walking to assess egocentric spatial memory (8.3%) (Dordevic et al., 2022), and one study used the Brief Visuospatial Memory Test-Revised (8.3%) (Tang et al., 2023).

As stated before, a smaller number of studies were conducted in clinical populations (4; 33.3%), including major depressive disorder, Alzheimer’s disease, schizophrenia, and clinical high-risk/first-episode psychosis (Demirtas-Tatlidede et al., 2010; Tang et al., 2023; Traikapi et al., 2023; Asl et al., 2024). Among these studies, three (25.0%) reported improved performance following stimulation. Specifically, unilateral rTMS improved performance on the Corsi Block-Tapping Test in patients with treatment-resistant depression, whereas bilateral stimulation did not produce significant effects (Asl et al., 2024). Similarly, iTBS improved performance on the Spatial Span task in patients with schizophrenia (Demirtas-Tatlidede et al., 2010), and rTMS improved performance on the Brief Visuospatial Memory Test-Revised in clinical high-risk and first-episode psychosis samples (Tang et al., 2023). In contrast, no significant improvement was observed in elderly adults with probable Alzheimer’s disease following rTMS, as assessed by the Corsi Block-Tapping Task (Traikapi et al., 2023).

The majority of studies were conducted on healthy adults (8; 66.7%) (Nilakantan et al., 2017; Marin et al., 2018; Guidali et al., 2019; Goldsworthy et al., 2020; Dordevic et al., 2022; Hermiller et al., 2022; Deng et al., 2024; Huang et al., 2024). Among studies using span-based tasks, two (16.6%) reported improved performance following stimulation (Deng et al., 2024; Huang et al., 2024), whereas one study (8.3%) reported impaired performance, reflected by increased serial order errors following stimulation (Guidali et al., 2019). Among studies using object–location memory paradigms, behavioral effects varied depending on stimulation protocol and experimental conditions. One study (8.3%) reported impaired performance following TBS, reflected by increased swap errors (Hermiller et al., 2022), and another study (8.3%) reported impaired long-term associative recognition and spatial recall under active retrieval conditions (Marin et al., 2018). In contrast, one study (8.3%) reported improved memory precision following stimulation (Nilakantan et al., 2017), while another study (8.3%) reported no significant changes in behavioral performance (Goldsworthy et al., 2020). Interestingly, the study using a Spatial Updating Paradigm via blind walking found that cTBS impaired egocentric spatial memory during dynamic spatial updating, reflected by an increase in distance and angular errors, with no effects observed in a static control condition (Dordevic et al., 2022).

### 3.4 Brain and neurophysiological outcomes

#### 3.4.1 Animal studies

This section summarizes the main neurobiological outcomes. Detailed study-level information is provided in Table 2.

Across the 23 included animal studies, 21 (91.3%) included brain-related measures, where the hippocampus was the most frequently assessed structure, either exclusively or in combination with cortical, limbic, or lesion-related regions.

Across the four studies which investigated the effects of rTMS on Alzheimer’s disease models (Tan et al., 2013; Wang et al., 2015; Huang et al., 2017; Bao et al., 2021), at the synaptic level, rTMS either fully or partially reversed Aβ_1–42_-induced suppression of hippocampal LTP (Tan et al., 2013; Wang et al., 2015; Huang et al., 2017), modulated cortical excitability, and restored the activity of large-conductance calcium-potassium channels (Wang et al., 2015). At the cellular and molecular level, Bao et al. (2021) reported reduced neuronal degeneration, necrosis and cell loss in cortex and hippocampus, as assessed via hematoxylin and eosin staining, together with a decrease in apoptotic neurons measured by terminal deoxynucleotidyl transferase–mediated dUTP nick end labeling (TUNEL). These effects were associated with reduced Bax expression and increased Bcl-2 expression in both regions, as well as reduced Caspase-3 expression in the cortex (Bao et al., 2021). Huang et al. (2017) reported reduced amyloidogenic processing of amyloid precursor protein (APP), as indicated by decreased BACE1, C99 and C89 levels. An increased expression of the activity-dependent scaffold protein Homer1a in transgenic was also found (Wang et al., 2015; Huang et al., 2017), as well as increased hippocampal neurotrophin expression, including BDNF and nerve growth factor (NGF) (Tan et al., 2013). Additionally, it was observed that rTMS increased protein levels of cAMP, PKAc and phosphorylated CREB protein levels in both regions (Bao et al., 2021) and up-regulated hippocampal N-methyl-D-aspartate (NMDA) receptor subunits NR1, NR2A, and NR2B (Tan et al., 2013).

Considering the three vascular dementia models (Yang et al., 2014, 2015; Zhang et al., 2015), rTMS attenuated hippocampal synaptic ultrastructural damage and enhanced CA3–CA1 LTP (Yang et al., 2015; Zhang et al., 2015). Additionally, rTMS up-regulated NMDA receptor (NMDAR1) and the anti-apoptotic protein Bcl-2 while down-regulating the pro-apoptotic protein Bax (Yang et al., 2015) and significantly increased mammalian target of rapamycin (mTOR) and eukaryotic initiation factor 4E (eIF-4E) protein expression, with comparable molecular effects observed following high- and low- frequency stimulation (Yang et al., 2014). Zhang et al. (2015) further demonstrated that rTMS enhanced CA3–CA1 LTP and up-regulated vascular endothelial growth factor (VEGF) expression, while restoring reduced levels of BDNF and NMDA receptor subunits NR1 and NR2B; NR2A expression remained unchanged.

Regarding stroke-related models (Hong et al., 2024; Jiang et al., 2025) or cerebral microinfarction (Lin et al., 2024), improvements were also observed. Hong et al. (2024) restored hippocampal synaptic ultrastructure, as shown by the increase in the length of the active zone, width of the synaptic cleft and, PSD thickness, in addition with increased hippocampal levels of postsynaptic density protein 95 (PSD95), synaptophysin (SYN) and BDNF expression. Jiang et al. (2025) reported reduced hippocampal apoptosis and enhanced axonal and myelin regeneration, associated with modulation of SARM1/HSP70/NF-κB signaling and regulation of Bax and Bcl-2 expression. In the cerebral microinfarction model, Lin et al. (2024) reported that cTBS enhanced paravascular cerebrospinal fluid–interstitial fluid exchange and cerebrospinal fluid infiltration into the brain parenchyma, as demonstrated by tracer studies. Stimulation also restored AQP4 expression and perivascular polarization, attenuated neuronal loss, and reduced astrocytic and microglial activation in lesion areas. Brain injury models showed increased hippocampal expression of BDNF, VEGF, and microtubule-associated protein 2 (MAP2) (Kim et al., 2024), while Qin et al. (2024) observed increased hippocampal neurogenesis, reflected by elevated DCX, Ki67, BrdU+/DCX+, and BrdU+/NeuN+ cells. rTMS also reduced microglial activation and pyroptosis markers and down-regulated NLRP3 inflammasome components. Additionally, stimulation increased PSD95, SYN, dendritic spine density, and activated BDNF–TrkB–CREB signaling.

In the psychiatric-related models rTMS modulated hippocampal plasticity and glial markers (Shang et al., 2019; Jiang et al., 2023). Jiang et al. (2023) reversed depressive- induced alterations in hippocampal CA3 astrocytic and neural progenitor markers, GFAP levels and nestin expression. Similarly, in the prenatal stress model, rTMS increased LTP at Schaffer collateral–CA1 synapses and up-regulated BDNF/TrkB and p-CREB/CREB signaling. Increased NR2A and NR2B expression and reduced apoptotic markers, reflected by an increased Bcl-xl/Bax ratio and decreased caspase-3 levels, were also reported (Shang et al., 2019). rTMS after sleep deprivation ameliorated synaptic and ultrastructural alterations, including increased dendritic spine density and synapse number. Transcriptomic analysis identified 56 differentially expressed genes, with KMO being significantly up-regulated in sleep-deprived rats and normalized following rTMS (Zheng et al., 2024).

In healthy animals, rTMS modulated hippocampal plasticity and network-level activity. Shang et al. (2016) reported increased short-term potentiation (STP), LTP, depotentiation, and paired-pulse facilitation in the CA1 region, together with increased hippocampal BDNF, SYN, and NR2B expression. Wu et al. (2022) reported up-regulation of NMDA receptor subunits (NR1, NR2A, NR2B) and activation of BDNF/TrkB/p-Akt signaling in prefrontal cortex and hippocampus, as well as increased regional homogeneity in cortico– striatal–thalamic and default mode network regions. In contrast, Zorzo et al. (2021) reported reduced cytochrome c oxidase (CCO) activity in limbic regions associated with spatial task performance.

Finally, in normally aged animals, rTMS modulated hippocampal synaptic ultrastructure and plasticity-related proteins in a stimulation-parameter-dependent manner. Ma et al. (2014) reported that 110% RMT increased synapse density, postsynaptic density thickness, and expression of SYN, GAP-43, PSD95, and BDNF/TrkB, whereas 150% RMT was associated with synaptic ultrastructural degeneration and reduced expression of plasticity-related markers. Ma et al. (2019) reported that 5 Hz stimulation increased postsynaptic density thickness, reduced synaptic cleft width, and up-regulated SYN, PSD95, and BDNF–pCREB signaling, whereas 25 Hz stimulation showed limited or reduced structural effects.

#### 3.4.2 Human studies

This section summarizes the main neurophysiological outcomes in human studies, focusing on electroencephalography (EEG) and functional magnetic resonance imaging (fMRI). Detailed study-level information is provided in Table 3.

Four of the twelve included human studies (4; 33.3%) used neurophysiological techniques to assess the effects of TMS (Nilakantan et al., 2017; Goldsworthy et al., 2020; Hermiller et al., 2022; Deng et al., 2024). Deng et al. (2024) reported that rTMS increased parietal alpha power during spatial memory maintenance and enhanced contralateral delay activity amplitude following stimulation, in line with their behavioral findings showing improvements in visuospatial short-term memory, both during stimulation (online) and after stimulation (offline). Nilakantan et al. (2017) found that rTMS reduced theta–alpha oscillatory activity and late positive event-related potential (ERP) amplitudes during recollection, with these neural changes being associated with improvements in memory precision. In contrast, Goldsworthy et al. (2020) reported no behavioral improvement in visuospatial memory following iTBS in older adults; however, in young adults, EEG measures revealed increased prefrontal cortical excitability, with an enhanced P60 TMS- evoked potential amplitude. Finally, Hermiller et al. (2022) employed fMRI to assess functional connectivity changes following stimulation. This study measured resting-state functional connectivity within the hippocampal network and reported increased connectivity following TBS in young adults. In contrast, 20 Hz rTMS did not produce significant connectivity changes. In older adults, TBS was associated with widespread connectivity alterations, whereas 20 Hz stimulation did not produce significant effects.

To note, among the 12 human studies included, eight (66.7%) did not include any neurophysiological measures (Demirtas-Tatlidede et al., 2010; Marin et al., 2018; Guidali et al., 2019; Dordevic et al., 2022; Tang et al., 2023; Traikapi et al., 2023; Asl et al., 2024; Huang et al., 2024).

## 4 Discussion

In this systematic review, we examined the available evidence on the effects of TMS on spatial memory across both animal and human studies. Tasks were operationally defined as spatial memory only when they required direct recall of spatial sequences or locations, excluding paradigms involving additional manipulation or decision processes (e.g., delayed recognition or match/non-match), which were classified as spatial working memory and therefore excluded from the analysis. Across the studies included in this review, TMS was applied using a range of stimulation protocols and cortical targets, and outcomes were assessed through both behavioral and neurophysiological measures.

### 4.1 Effects of TMS on spatial memory in health

In healthy participants, TMS is primarily used as an experimental tool to probe the causal contribution of specific cortical regions to spatial memory processes and their interaction with hippocampal-dependent representations. A comparable variability in behavioral outcomes was observed across both animal models and healthy human participants, with the direction of TMS effects differing depending on stimulation parameters and experimental conditions. In animal models, several studies reported improvements in spatial learning and memory performance following stimulation, reflected by reduced escape latency and increased time spent in the target quadrant or platform crossings both in adults and normally aged subjects (Ma et al., 2014, 2019; Shang et al., 2016; Wu et al., 2022). However, other studies reported impairments in spatial memory performance (Li et al., 2007; Ma et al., 2014; Zhang et al., 2024) or no significant behavioral changes (Zorzo et al., 2021). A similar heterogeneity was observed in healthy human studies, with stimulation producing improvements (Nilakantan et al., 2017; Deng et al., 2024; Huang et al., 2024), impairments (Marin et al., 2018; Guidali et al., 2019; Dordevic et al., 2022; Hermiller et al., 2022), or no measurable behavioral effects (Marin et al., 2018; Goldsworthy et al., 2020; Hermiller et al., 2022). Beyond spatial memory, TMS has also been investigated across other cognitive domains, with generally mixed results. In healthy adults, a quantitative meta-analysis of offline rTMS to the DLPFC found only small, stimulation-direction-specific effects on executive functioning, episodic memory, and visual perception, with no significant effect on working memory (Patel et al., 2020), whereas a more recent meta-analysis targeting the same region reported that rTMS may improve cognition overall relative to control conditions, albeit with considerable variability across studies (Ebrahimzadeh et al., 2025). This variability may be partly explained by differences in task demands. In humans, span-based paradigms—primarily engaging short-term maintenance processes—suggest that modulation of parietal networks can influence the online retention of spatial information, with some protocols leading to improved performance (Deng et al., 2024; Huang et al., 2024), while others disrupt serial order processing (Guidali et al., 2019; Dordevic et al., 2022). In contrast, object–location associative paradigms, which rely more heavily on hippocampal- dependent binding and relational memory, yield more heterogeneous outcomes. These include both improvements in memory precision (Nilakantan et al., 2017), in associative recognition and spatial recall, particularly under conditions that engage active retrieval processes (Marin et al., 2018; Hermiller et al., 2022), as well as null behavioral effects despite evidence of stimulation-induced plasticity, especially in older adults (Goldsworthy et al., 2020).

Beyond task demands, variability across studies is largely attributable to differences in stimulation parameters, including pulse number, frequency, intensity, treatment duration, and targeting strategies. All animal studies followed some kind of TMS protocol, except (Lin et al., 2024), which employed TBS. Although low-frequency stimulation (≤1 Hz) has traditionally been associated with reduced cortical excitability and higher-frequency (>1 Hz) stimulation with facilitatory effects, this classification mainly derives from motor- cortex physiology (Fitzgerald et al., 2006; Du et al., 2019), and should not be directly extrapolated to complex cognitive outcomes such as spatial memory. Consistent with this caution, the studies reviewed here did not show a simple relationship between stimulation frequency and behavioral outcome. Low-frequency protocols were often associated with improved spatial memory (Tan et al., 2013; Ma et al., 2014; Yang et al., 2014, 2015; Hong et al., 2024; Kim et al., 2024; Zheng et al., 2024). Conversely, behavioral impairments were not restricted to low-frequency stimulation and were also reported under high- frequency protocols (Li et al., 2007; Zhang et al., 2024). A particularly illustrative example is provided by Ma et al. (2014), where the same stimulation frequency (1 Hz) produced opposite effects depending on intensity: stimulation at 110% RMT improved spatial memory and enhanced hippocampal synaptic markers, whereas stimulation at 150% RMT worsened performance and was associated with synaptic degeneration and signs of neuronal damage. These findings indicate that frequency alone does not determine the direction of TMS effects, suggesting that canonical assumptions are insufficient to explain the modulation of spatial memory. Interpretation of stimulation effects is further complicated by substantial variability in intensity calibration procedures across studies. In animal studies, intensity was reported using different metrics, including MT–based dosing, percentages of maximum stimulator output, and magnetic field strength, whereas human studies predominantly expressed intensity relative to MT. Moreover, threshold determination methods varied across studies, ranging from motor- evoked potential–based approaches to visual inspection of evoked movements or muscle contractions. Such methodological variability limits direct comparisons across protocols and may contribute to the heterogeneity of behavioral and neurophysiological outcomes. Behavioral outcomes instead appear to emerge from the interaction between stimulation parameters and circuit-level factors. These discrepancies may arise because behavioral outcomes are not merely products of local excitability changes, but reflect broader network-level dynamics in which the functional impact of stimulation depends on the recruitment of distributed neural circuits and the baseline state of the system (Hunsaker and Kesner, 2018; Jannati et al., 2023). In line with mechanistic accounts (Ridding and Rothwell, 2007; Ridding and Ziemann, 2010; Hamada et al., 2013), this variability may reflect differential recruitment of interneuronal circuits and neuronal populations by the induced electric field, modulated by the baseline state of the network. This perspective is particularly relevant for spatial memory, which depends on coordinated hippocampal– extra-hippocampal interactions rather than local cortical excitability alone. Accordingly, the present evidence supports a state- and network-dependent framework, in which TMS may normalize dysfunctional circuits in pathological models while producing more variable effects in intact systems.

In addition to differences in frequency, variability in stimulation structure and delivery further contributes to heterogeneity in outcomes. Across animal studies, stimulation protocols showed marked variability, and key parameters such as session duration were not always reported. When specified, daily session durations were typically 1–12 min and interventions ranged from single-day stimulation (Li et al., 2007) to repeated stimulation schedules across several days (Ma et al., 2014, 2019; Shang et al., 2016; Zorzo et al., 2021; Wu et al., 2022; Zhang et al., 2024). Most studies employed once-daily stimulation using multi-train designs, although some implemented shorter or more intensive protocols. Notably, stimulation was often applied broadly over the skull rather than targeting specific cortical regions, potentially limiting spatial specificity. In human studies, although experimental designs were relatively more standardized, substantial variability remained in protocol implementation. Most experiments involved single- session stimulation designs delivered within one experimental day (Marin et al., 2018; Guidali et al., 2019; Goldsworthy et al., 2020; Dordevic et al., 2022; Hermiller et al., 2022; Huang et al., 2024), although a smaller number applied multi-day interventions (Nilakantan et al., 2017; Deng et al., 2024). Within single-session designs, the number of pulses ranged from ≤600 to over 2,000, and stimulation structure varied between multi- train, single-train, or continuous protocols, with some studies combining approaches or not reporting these parameters.

Importantly, variability in stimulation structure is closely linked to differences in target engagement. In human studies, stimulation most frequently targets parietal and prefrontal regions implicated in visuospatial processing (Marin et al., 2018; Guidali et al., 2019; Hermiller et al., 2022; Deng et al., 2024). Because these regions support distinct cognitive components of spatial memory, their modulation is likely to produce different behavioral effects. From a systems-level perspective, spatial memory depends on a distributed network in which the hippocampus supports the encoding of spatial and contextual information, posterior parietal regions contribute to spatial representation and integration, and prefrontal regions support higher-order control processes such as strategy selection and temporal organization (Hunsaker and Kesner, 2018). These regions operate as part of a broader network—including retrosplenial, entorhinal, and thalamic structures, among others—that supports the formation and maintenance of spatial representations (Hunsaker and Kesner, 2018; Zorzo et al., 2022; Torres-Morales and Cansino, 2024). However, it is important to note that, due to methodological constraints inherent to rodent TMS, stimulation in animal studies was predominantly applied over the skull without precise cortical targeting, resulting in relatively diffuse engagement of neural circuits. Even in studies attempting regional targeting (Zorzo et al., 2021; Wu et al., 2022), spatial specificity remains limited by coil-to-brain size ratio and scaling constraints.

Within this framework, differences in target engagement are expected to influence how TMS modulates distributed spatial memory networks at the neural level. In healthy animal models, stimulation has been associated with changes in synaptic plasticity and neurotrophic signaling, including increases in LTP, SYN short-term plasticity, and the expression of plasticity-related markers such as BDNF, and NMDA receptor subunits (Shang et al., 2016; Wu et al., 2022). These effects indicate that TMS can influence hippocampal and cortico-hippocampal circuits involved in the encoding and stabilization of spatial representations, as in these studies, such neural changes were accompanied by improvements in spatial memory performance. At a broader scale, stimulation has also been shown to modulate network-level dynamics, including alterations in functional connectivity within cortico–striatal–thalamic and default mode–like networks (Wu et al., 2022) indicating that its effects extend beyond local synaptic mechanisms.

Importantly, neural modulation does not always translate into measurable behavioral changes. For example, changes in brain metabolism, such as reduced CCO in limbic regions, have been reported in the absence of performance differences (Zorzo et al., 2021), potentially reflecting more efficient recruitment of neural resources. In addition, evidence from aging models indicates that stimulation attenuated spatial memory impairment associated with age, as shown in a reduction in escape latency and increased platform crossings, with greater improvements at 5 than 25 Hz. At the neural level, these effects were associated with enhanced synaptic thickness and up-regulation of neuroplasticity-related genes, suggesting a restoration of age-related deficits in hippocampal function (Ma et al., 2019). Similarly, others reported that behavioral outcomes depended critically on stimulation intensity, with the improvements observed at 110% RMT reflecting in increased synapse density and increased the expression of neuroplasticity-related genes, while impairments at 150% RMT correlated with synaptic ultrastructural degeneration and signs of neuronal damage and apoptosis (Ma et al., 2014). These findings may indicate that excessive stimulation intensity can induce maladaptive plasticity or neural stress, particularly in vulnerable aging systems. Together, these results highlight the sensitivity of the aging brain to stimulation parameters and emphasize the importance of optimal dosing for achieving beneficial cognitive and neurobiological effects.

In human studies, although neurophysiological evidence remains more limited, available findings converge with preclinical data in showing that TMS modulates distributed network activity. EEG studies have demonstrated changes in oscillatory dynamics associated with spatial memory processes, including increased parietal alpha power during memory maintenance and modulation of event-related potentials linked to memory encoding and retrieval (Nilakantan et al., 2017; Deng et al., 2024). In some cases, these neural changes are accompanied by improvements in behavioral performance, suggesting a functional role of oscillatory modulation. However, stimulation does not consistently enhance spatial memory, despite measurable neural effects (Goldsworthy et al., 2020). For instance, increases in prefrontal cortical excitability were observed in younger individuals, whereas in older adults behavioral performance was associated with stimulation-induced plasticity without clear performance gains. This pattern suggests that the effects of TMS may be expressed differently at the neural and behavioral levels depending on the physiological state of the system, and that neural modulation does not necessarily translate into immediate cognitive gains (Ridding and Ziemann, 2010; Hamada et al., 2013). More broadly, accumulating evidence suggests that the effects of non-invasive brain stimulation are highly state-dependent and shaped by interacting physiological and methodological factors, including age, baseline excitability, prior neural activity, network organization, and stimulation parameters (Silvanto et al., 2008; Zhang H. et al., 2025).

A similar dissociation between neural and behavioral effects is also observed in connectivity-based studies. Stimulation-induced changes in hippocampal network connectivity have been reported alongside either absent or even detrimental behavioral outcomes, and with qualitatively different patterns across age groups (Hermiller et al., 2022). In this context, increases in connectivity were accompanied by impaired performance in younger individuals, whereas in older adults, stimulation produced more diffuse and less specific network changes without clear behavioral effects. Thus, TMS may induce reorganization of large-scale memory networks that is not necessarily aligned with improved task performance, and whose functional impact depends on the baseline organization and plasticity of the system (Ridding and Ziemann, 2010). However, given the limited number of studies in healthy participants combining behavioral and neurophysiological measures, it remains unclear whether such network-level changes reflect facilitation, disruption, or compensatory reorganization of spatial memory processes. Future studies integrating behavioral, connectivity, and neurophysiological measures will be critical to determine whether these network-level modulations reflect facilitation, disruption, or compensatory reorganization of spatial memory processes.

### 4.2 Effects of TMS on spatial memory in pathological conditions

In pathological contexts, TMS is primarily applied with a therapeutic purpose, aiming to alleviate spatial disorientation and memory deficits. In these conditions, stimulation provides a framework to examine how externally driven modulation of cortical excitability interacts with disrupted hippocampal–cortical circuits underlying spatial memory.

Across pathological models, TMS was generally associated with improvements in spatial memory performance, although the magnitude and consistency of these effects varied across conditions. In animal models, rTMS was most consistently associated with improvements in spatial memory performance in neurodegenerative and vascular conditions: Alzheimer’s disease-related models reported attenuation of spatial learning and memory deficits following stimulation (Tan et al., 2013; Wang et al., 2015; Huang et al., 2017; Bao et al., 2021), and models of vascular dementia similarly showed recovery of spatial learning and memory performance (Yang et al., 2014, 2015; Zhang et al., 2015). Beneficial effects were also reported in models of cerebral ischemia and stroke (Hong et al., 2024; Lin et al., 2024; Jiang et al., 2025), as well as in traumatic or radiation- induced brain injury models (Kim et al., 2024; Qin et al., 2024). Similarly, models of psychiatric and stress-related conditions showed beneficial effects of stimulation on spatial memory performance (Shang et al., 2019; Jiang et al., 2023), and rTMS was also reported to attenuate spatial learning deficits induced by sleep deprivation (Zheng et al., 2024). A comparable, albeit more limited, pattern was observed in clinical populations. TMS was associated with improvements in visuospatial memory performance in several conditions, including treatment-resistant depression, schizophrenia, and clinical high-risk or first-episode psychosis (Demirtas-Tatlidede et al., 2010; Tang et al., 2023; Asl et al., 2024). However, no significant improvement was observed in elderly adults with probable Alzheimer’s disease following stimulation (Traikapi et al., 2023). Although the findings in clinical populations are encouraging, they should be interpreted with caution, since only four studies investigated clinical populations. Additionally, sample sizes were generally small, particularly in the Alzheimer’s disease study (Traikapi et al., 2023). As such, conclusions regarding the therapeutic relevance of TMS for spatial memory in clinical contexts remain preliminary and warrant replication in larger, more adequately powered trials.

Overall, evidence from both animal models and clinical populations suggests that TMS exerts more consistent facilitatory effects on spatial memory under pathological conditions than in healthy systems. This pattern should not be interpreted as a uniform pro-cognitive effect, but rather as reflecting a state-dependent mechanism of action, whereby stimulation interacts with ongoing brain activity and pre-existing network dysfunction (Silvanto et al., 2008; Sack et al., 2024). In disrupted systems, characterized by altered excitability, impaired plasticity, or aberrant connectivity, TMS may facilitate the normalization or rebalancing of network dynamics, thereby enabling measurable behavioral improvements. Given heterogeneity in disease progression and underlying neuropathology, the efficacy of TMS may depend less on the stimulation protocol per se than on its interaction with the functional state of the targeted circuits. This framework may account for the more consistent effects observed in preclinical models, where pathology and experimental conditions are tightly controlled, whereas evidence in clinical populations remains limited and less systematically characterized, constraining mechanistic interpretation.

However, despite the relatively controlled nature of preclinical models, stimulation parameters across animal studies showed substantial variability in pulse number, frequency, and treatment duration. Beyond differences in dosing, a key methodological limitation concerns spatial specificity. In most studies, stimulation was delivered broadly over the skull or dorsal midline rather than targeting specific cortical regions (Tan et al., 2013; Yang et al., 2014, 2015; Zhang et al., 2015; Wang et al., 2015; Huang et al., 2017; Bao et al., 2021; Jiang et al., 2023, 2025; Zheng et al., 2024; Lin et al., 2024; Qin et al., 2024), with only a minority applying more focal stimulation to parietal or frontal areas (Shang et al., 2016; Hong et al., 2024; Kim et al., 2024). This limited spatial resolution reflects fundamental constraints imposed by the coil-to-brain size ratio in rodent models, resulting in diffuse activation of neural tissue and reduced control over target engagement. As a result, non-focal stimulation may recruit widespread and heterogeneous neural populations, complicating the attribution of behavioral effects to specific circuits underlying spatial memory. This issue is particularly relevant given the distributed organization of hippocampal–cortical networks supporting spatial representations (Hunsaker and Kesner, 2018), where the selective engagement of functionally relevant pathways is likely a key determinant of stimulation efficacy. Notably, recent methodological advances have demonstrated that more focal stimulation can be achieved in awake rodents using coil-guided targeting approaches. In this context, focal TMS has been shown to induce spatially restricted increases in neuronal activity, as indexed by enhanced glucose uptake within confined cortical regions, with an effect in deep cortical layers (Cermak et al., 2020), highlighting that improving spatial precision through coil design and targeting strategies is a key determinant of neural engagement and may substantially enhance the translational relevance of preclinical TMS models. In this regard, although hippocampal involvement remains plausible given that all outcomes were assessed through spatial memory tasks, the diffuse nature of most stimulation protocols makes it difficult to determine whether the reported synaptic and molecular changes reflect direct local engagement of hippocampal circuits or indirect network-level modulation reaching this structure from co-localized regions.

In clinical studies, stimulation protocols differed in session structure and treatment schedules, ranging from single-session designs to short multi-day interventions (Demirtas-Tatlidede et al., 2010; Tang et al., 2023; Traikapi et al., 2023; Asl et al., 2024). Stimulation targets included prefrontal, parietal, cerebellar, and posterior medial regions, specifically the dorsolateral prefrontal cortex, inferior parietal lobule, cerebellar vermis, and precuneus. These targets largely correspond to regions embedded within large-scale networks supporting cognitive control and spatial processing, rather than being specifically optimized for hippocampal-dependent spatial memory. In this context, differences in behavioral outcomes are likely to reflect the interaction between stimulation parameters, cortical target, and disorder-specific network alterations, as emphasized by current TMS frameworks. Notably, the study conducted in treatment- resistant depression (Asl et al., 2024) stimulation over the dorsolateral prefrontal cortex followed a frequency-dependent bilateral protocol (high-frequency left, low- frequency right) consistent with established TMS frameworks (Lefaucheur et al., 2014; Rossi et al., 2021). This suggests that, even when the aim is to modulate cognitive functions such as spatial memory, stimulation parameters are frequently guided by clinically validated TMS frameworks rather than protocols specifically optimized for memory processes.

At the neural level, evidence from animal models indicates that the effects of TMS in pathological conditions are mediated by converging mechanisms acting across synaptic, cellular, and network levels. Across disease models, improvements in spatial memory are consistently accompanied by restoration of hippocampal plasticity and engagement of neurotrophic signaling pathways (Shang et al., 2016; Bao et al., 2021; Qin et al., 2024).

Interestingly, the beneficial effects of TMS on spatial memory in Alzheimer’s disease models are consistently accompanied by convergent changes in hippocampal plasticity and neurotrophic signaling. Across studies, attenuation of spatial learning and memory deficits was associated with restoration of synaptic function, modulation of cortical excitability, and engagement of molecular pathways involved in neurodegeneration and neuronal survival (Tan et al., 2013; Wang et al., 2015; Huang et al., 2017; Ma et al., 2017; Bao et al., 2021). Notably, TMS was associated with improvements in hippocampal plasticity and attenuation of amyloid-related pathology, as reflected by reduced Aβ and recovery of activity-dependent synaptic function (Tan et al., 2013; Wang et al., 2015; Huang et al., 2017). These findings suggest that stimulation may counteract key mechanisms underlying synaptic dysfunction in Alzheimer’s disease models, thereby facilitating spatial learning and memory processes. Importantly, these effects did not map onto a simple low- versus high-frequency distinction. While 1 Hz TMS improved spatial memory and restored hippocampal plasticity in Aβ-related models (Tan et al., 2013; Huang et al., 2017), beneficial effects were also reported with higher-frequency protocols, including 5 Hz in SAMP8 mice (Bao et al., 2021) and, notably, 15 Hz in 3xTg mice, where 1 Hz and 10 Hz did not produce comparable behavioral improvements (Wang et al., 2015). In parallel, magnetic stimulation modulated key intracellular signaling pathways, including restoration of the cAMP/PKA/CREB cascade and increased expression of neurotrophic factors such as BDNF and NGF (Tan et al., 2013; Bao et al., 2021). These changes are consistent with activity-dependent molecular programmes known to support long-term memory stabilization, including CREB- and BDNF-mediated signaling (Sun et al., 2024). Moreover, it has been found TMS reduced neuronal degeneration, apoptosis, and synaptic dysfunction in hippocampal and cortical regions (Wang et al., 2015; Bao et al., 2021), by normalization of calcium homeostasis, attenuation of excitotoxicity, and restoration of excitatory–inhibitory balance. Together, these findings support a model in which TMS promotes cognitive recovery in Alzheimer’s through coordinated modulation of synaptic plasticity, neurotrophic signaling, and neuroprotective processes. These findings are in line with recent integrative accounts of rTMS mechanisms in Alzheimer’s disease, which similarly emphasize its multilevel effects across synaptic, cellular, and network domains (Antonioni et al., 2025).

Similarly, in vascular dementia models, the beneficial effects of rTMS on spatial memory were consistently associated with restoration of hippocampal plasticity and engagement of molecular pathways involved in synaptic function and neuronal survival (Yang et al., 2014, 2015; Zhang et al., 2015). Across studies, improvements in spatial learning and memory were accompanied by recovery of CA3–CA1 LTP, attenuation of synaptic ultrastructural damage, and increased expression of plasticity-related proteins, including BDNF and NMDAR subunits (Yang et al., 2015; Zhang et al., 2015). In parallel, rTMS modulated intracellular signaling pathways linked to protein synthesis and synaptic remodeling, such as mTOR and eIF4E (Yang et al., 2014), suggesting a potential role in supporting long-term memory consolidation. These findings suggest that rTMS efficacy in vascular dementia is linked to its capacity to recruit plasticity and survival-related mechanisms in compromised hippocampal networks. In addition, rTMS was associated with modulation of apoptotic pathways, including increased Bcl-2 and reduced Bax expression (Yang et al., 2015) pointing to a shift toward neuronal survival in ischemia- vulnerable hippocampal circuits.

Beneficial effects were consistently reported across models of cerebral ischemia, stroke, and brain injury, where improvements in spatial memory were accompanied by coordinated structural, cellular, and molecular changes (Hong et al., 2024; Lin et al., 2024; Qin et al., 2024; Jiang et al., 2025). At the structural level, rTMS restored hippocampal synaptic ultrastructure and function, promoted axonal and myelin regeneration, and reduced neuronal loss within lesion areas. These changes were paralleled by increased expression of neurotrophic and plasticity-related markers, including BDNF, VEGF, and MAP2, as well as enhanced hippocampal neurogenesis, indicating activation of regenerative processes following neural damage. Importantly, these effects do not appear to arise from isolated mechanisms but rather from the convergence of multiple interacting processes, among which neuroinflammation emerges as a central target. Across studies, rTMS attenuated astrocytic and microglial activation and suppressed pro-inflammatory and pyroptotic signaling pathways, including NLRP3 inflammasome activation and downstream mediators such as caspase-1 and IL-1β (Lin et al., 2024; Qin et al., 2024). Given that excessive glial activation contributes to secondary neuronal damage, synaptic dysfunction, and disruption of hippocampal connectivity after vascular and radiation injury, these findings suggest that TMS may facilitate cognitive recovery partly by limiting maladaptive inflammatory cascades. In parallel, rTMS also regulated apoptosis-related pathways, including SARM1-mediated HSP70/NF-κB signaling, shifting the balance toward cell survival and tissue repair (Jiang et al., 2025). Notably, converging evidence indicates that these neuroprotective and reparative effects are mechanistically linked to activity-dependent neurotrophic signaling. In particular, findings from irradiation-induced injury models demonstrate that pharmacological blockade of BDNF signaling abolishes both behavioral improvements and associated neurobiological changes, providing causal evidence for its central role (Qin et al., 2024). This suggests that neurotrophic pathways may act as integrative hubs through which rTMS coordinates synaptic plasticity, neurogenesis, and inflammatory regulation. In this framework, rTMS may not only mitigate neural damage but also promote a permissive microenvironment that supports synaptic remodeling, network reorganization, and hippocampal-dependent learning. These effects were consistently observed following repeated stimulation protocols, indicating that cumulative neuromodulatory influences are required to engage slow biological processes such as structural remodeling, glioregulation, and neurogenesis. Consistent with this interpretation, clinical evidence in stroke populations shows that rTMS modulates cortical excitability in a state-dependent manner, contributing to the rebalancing of large-scale network dynamics rather than exerting uniform excitatory or inhibitory effects (Bai et al., 2022). Although these studies do not directly assess spatial memory, they support the translational relevance of network- level plasticity mechanisms observed in preclinical models.

Finally, in the psychiatric-related models, the behavioral improvements induced by rTMS were associated with restoration of hippocampal plasticity, glial homeostasis, and cell survival mechanisms (Shang et al., 2019; Jiang et al., 2023). Chronic and prenatal stress paradigms were characterized by impairments in CA3–CA1 LTP, neuronal integrity, astrocytic support, and neural stem cell dynamics, all of which are critical for hippocampal-dependent memory processes. rTMS reversed these alterations, primarily through activation of BDNF/TrkB signaling and re-engagement of NMDA- and CREB- dependent plasticity pathways, supporting a central role for activity-dependent neurotrophic modulation in restoring synaptic efficacy (Shang et al., 2016). Notably, normalization of astrocytic and progenitor markers (e.g., GFAP, nestin) further indicates recovery of hippocampal microenvironment homeostasis (Jiang et al., 2023), which is essential for metabolic support, neurotransmitter regulation, and synaptic stability. Stress- induced astrocytic loss is known to disrupt metabolic support, neurotransmitter buffering, and synaptic regulation, and thus contribute to impaired plasticity and neuronal vulnerability. By restoring astrocytic populations and promoting neural stem cell differentiation toward mature glial phenotypes, rTMS may facilitate structural and functional repair of hippocampal circuits. In parallel, the reduction in neuronal loss and apoptotic signaling observed after stimulation suggests that these changes contribute to increased cellular survival and function. These findings extend the mechanistic framework derived from neurodegenerative and injury models by demonstrating that similar neurotrophic and plasticity-related processes operate in stress-induced dysfunction, where alterations are primarily network- and microenvironment-driven rather than structurally induced. Together, this evidence supports a unified model in which rTMS counteracts cognitive dysfunction through integrated modulation of neurotrophic signaling, synaptic plasticity, glial regulation, and cellular resilience (Shang et al., 2019; Jiang et al., 2023).

In clinical populations, none of the studies included in this review directly assessed neurophysiological or brain-level changes associated with TMS, limiting interpretation to behavioral outcomes. Although several studies reported improvements in spatial memory in conditions such as depression, schizophrenia, and early psychosis (Demirtas-Tatlidede et al., 2010; Tang et al., 2023; Traikapi et al., 2023; Asl et al., 2024), the absence of concurrent neural measures precludes determining whether these effects reflect restoration of hippocampal function, compensatory engagement of fronto-parietal networks, or more general modulation of large-scale brain dynamics. Consequently, mechanistic interpretations in humans must remain tentative and are currently informed primarily by preclinical evidence, where behavioral improvements are consistently linked to plasticity-related processes. This gap highlights an important limitation in the literature and points to the need for integrative approaches combining behavioral and neurophysiological assessments to better characterize the mechanisms underlying TMS effects in clinical populations. Beyond spatial memory, TMS effects on other cognitive domains in clinical, cross-diagnostic populations are similarly modest and domain- specific: a large-scale meta-analysis of left DLPFC rTMS across neuropsychiatric disorders reported small but significant effects on global cognition, declarative memory, working memory, and cognitive control, alongside non-significant effects on attention and language (Kan et al., 2023).

It is also worth noting that the psychiatric and neurodegenerative evidence bases within this review are asymmetric: three clinical studies addressed psychiatric conditions (Demirtas-Tatlidede et al., 2010; Tang et al., 2023; Asl et al., 2024), whereas only one addressed a neurodegenerative disorder(Alzheimer’s disease; Traikapi et al., 2023), employing a single-case, multiple-baseline design with four participants. This limits the extent to which the null finding in this study can be generalized to TMS efficacy in neurodegenerative populations more broadly, and future work should prioritize larger controlled trials in this population. Furthermore, in the three clinical studies reporting medication status, TMS was consistently delivered as an adjunct to stable pharmacological treatment rather than as a standalone intervention (Demirtas-Tatlidede et al., 2010; Tang et al., 2023; Traikapi et al., 2023), representing a further source of variability not addressed by the included evidence.

## 5 Conclusion

The evidence reviewed indicates that TMS is capable of modulating spatial memory and its underlying neural substrates across both animal and human studies, although its effects are variable and dependent on stimulation parameters, task demands, and the physiological state of the system.

There is, however, an important distinction between the animal and human literature that should be noted. Animal studies were primarily designed to assess the biological mechanisms underlying TMS-induced cognitive changes, combining behavioral assessments with molecular, cellular, and histological analyses to characterize synaptic plasticity, neurotrophic signaling, apoptosis, and neuroinflammatory processes. In contrast, human studies were mainly focused on evaluating behavioral outcomes and potential clinical applications, with comparatively limited investigation of the underlying neurophysiological mechanisms, owing to the inherent constraints of non-invasive measurement approaches available in human research.

In healthy conditions, TMS produces heterogeneous behavioral outcomes, reflecting the complexity of modulating intact hippocampal–cortical networks and the sensitivity of spatial memory processes to task-specific and network-level dynamics. In contrast, under pathological conditions, stimulation more consistently facilitates spatial memory performance, likely by interacting with disrupted circuits and promoting the reorganization or normalization of large-scale network activity. This asymmetry suggests that the effects of TMS are not inherently pro-cognitive, but rather emerge from state- dependent interactions with ongoing neural activity. In intact systems, stimulation may perturb or transiently reconfigure network dynamics, whereas in pathological conditions it may restore altered excitability, plasticity, and connectivity, leading to more consistent behavioral improvements. Across studies, the effects of TMS are consistently associated with changes in synaptic plasticity, neurotrophic signaling, neuroinflammation, and cellular survival processes. These processes appear to operate in an integrated and state- dependent manner, with neurotrophic signaling playing a central role in linking cellular changes to behavioral outcomes.

Despite these advances, several limitations should be considered when interpreting the current evidence. Substantial heterogeneity exists across studies in stimulation parameters, including frequency, intensity, pulse number, treatment duration, and targeting strategies, as well as in the behavioral paradigms used to assess spatial memory. It is also worth noting that the MWM was the predominant paradigm across animal studies(21 of 23), and performance in this task can be influenced by factors beyond spatial memory itself, such as swimming ability or stress reactivity (Vorhees and Williams, 2006). Dry-land alternatives, such as the Barnes maze or Y-maze, were used far less frequently (one study each), and their broader incorporation in future work could help corroborate findings across testing modalities. In addition, important methodological differences between animal and human studies complicate direct translational comparisons and limit circuit-specific mechanistic attribution: animal studies frequently relied on non-focal, whole-skull or dorsal midline stimulation rather than region-specific targeting, whereas human studies, including those in pathological and clinical populations, targeted distinct cortical regions (e.g., dorsolateral prefrontal, parietal, cerebellar, temporoparietal). Few studies, even in clinical populations, assessed outcomes beyond the immediate post-stimulation period; future work should incorporate longitudinal designs with repeated behavioral and neurophysiological assessments to establish treatment durability. Furthermore, translation to humans remains limited because few studies combine behavioral and neurophysiological measures, especially in clinical populations, and outcome measures relied mainly on standardized behavioral indices.

These limitations restrict mechanistic interpretation and highlight the need for integrative approaches that simultaneously assess cognitive performance, network dynamics, and physiological responses to stimulation. Future research should prioritize the systematic characterization of stimulation parameters, the development of more precise targeting strategies, and the incorporation of multimodal outcome measures to better capture the interaction between TMS and distributed memory networks. Taken together, these findings highlight that the efficacy of TMS on spatial memory depends on aligning stimulation parameters with the functional state and network organization of the brain, underscoring the importance of individualized, circuit-informed approaches to optimize its therapeutic potential in disorders affecting spatial cognition.

A key challenge for the field is identifying which individuals are most likely to benefit from TMS-based interventions targeting spatial memory. Current evidence suggests that therapeutic effects are most consistent in pathological conditions characterized by disrupted hippocampal–cortical circuits, yet substantial variability exists even within clinical populations, likely reflecting differences in disease stage, baseline network integrity, and individual neurophysiological characteristics. Advances in neuroimaging- guided targeting and the use of individual functional connectivity profiles to personalize stimulation sites represent promising strategies to improve patient stratification and treatment precision (Gao et al., 2025; Zhang W. et al., 2025). Beyond protocol optimization, there is growing interest in combining TMS with complementary therapeutic approaches to augment its effects. Pairing TMS with cognitive training or memory rehabilitation tasks during or immediately after stimulation may leverage stimulation-induced increases in cortical excitability and synaptic plasticity to enhance learning-related consolidation, an approach that has already shown larger cognitive benefits than TMS alone in post-stroke cognitive impairment and in Alzheimer’s disease and mild cognitive impairment (Gao et al., 2023; Yang et al., 2024). Similarly, combining TMS with pharmacological agents that modulate neurotrophic or glutamatergic signaling — pathways consistently engaged by TMS across the studies reviewed here — could potentiate and prolong its effects on hippocampal-dependent memory. These combinatorial approaches remain largely unexplored in the context of spatial memory specifically, and represent a high-priority direction for future translational research.

## Data Availability

The original contributions presented in this study are included in this article/supplementary material, further inquiries can be directed to the corresponding authors.
